# *Peli3* ablation ameliorates acetaminophen-induced liver injury through inhibition of GSK3β phosphorylation and mitochondrial translocation

**DOI:** 10.1038/s12276-023-01009-w

**Published:** 2023-06-01

**Authors:** Jaewon Lee, Jihoon Ha, Jun-Hyeong Kim, Dongyeob Seo, Minbeom Kim, Yerin Lee, Seong Shil Park, Dahee Choi, Jin Seok Park, Young Jae Lee, Siyoung Yang, Kyung-Min Yang, Su Myung Jung, Suntaek Hong, Seung-Hoi Koo, Yong-Soo Bae, Seong-Jin Kim, Seok Hee Park

**Affiliations:** 1 Department of Biological Sciences, Sungkyunkwan University, Suwon, 16419 Republic of Korea; 2 Department of Life Science, Korea University, Seoul, 02841 Republic of Korea; 3 Department of Biochemistry, Gachon University School of Medicine, Incheon, 21999 Republic of Korea; 4 Department of Pharmacology, Ajou University School of Medicine, Suwon, 16499 Republic of Korea; 5 SRC Center for Immune Research on Non-lymphoid Organs, Sungkyunkwan University, Suwon, 16419 Republic of Korea; 6 Medpacto Inc., Seoul, 06668 Republic of Korea; 7 GILO Institute, GILO Foundation, Seoul, 06668 Republic of Korea; 8 Present Address: KoBio Labs, Seongnam, 13488 Republic of Korea

**Keywords:** Ubiquitylation, Experimental models of disease

## Abstract

The signaling pathways governing acetaminophen (APAP)-induced liver injury have been extensively studied. However, little is known about the ubiquitin-modifying enzymes needed for the regulation of APAP-induced liver injury. Here, we examined whether the Pellino3 protein, which has E3 ligase activity, is needed for APAP-induced liver injury and subsequently explored its molecular mechanism. Whole-body *Peli3*^−/−^ knockout (KO) and adenovirus-mediated *Peli3* knockdown (KD) mice showed reduced levels of centrilobular cell death, infiltration of immune cells, and biomarkers of liver injury, such as alanine aminotransferase (ALT) and aspartate aminotransferase (AST), upon APAP treatment compared to wild-type (WT) mice. *Peli3* deficiency in primary hepatocytes decreased mitochondrial and lysosomal damage and reduced the mitochondrial reactive oxygen species (ROS) levels. In addition, the levels of phosphorylation at serine 9 in the cytoplasm and mitochondrial translocation of GSK3β were decreased in primary hepatocytes obtained from *Peli3*^−/−^ KO mice, and these reductions were accompanied by decreases in JNK phosphorylation and mitochondrial translocation. Pellino3 bound more strongly to GSK3β compared with JNK1 and JNK2 and induced the lysine 63 (K63)-mediated polyubiquitination of GSK3β. In rescue experiments, the ectopic expression of wild-type Pellino3 in *Peli3*^−/−^ KO hepatocytes restored the mitochondrial translocation of GSK3β, but this restoration was not obtained with expression of a catalytically inactive mutant of Pellino3. These findings are the first to suggest a mechanistic link between Pellino3 and APAP-induced liver injury through the modulation of GSK3β polyubiquitination.

## Liver injury: Damage from common painkiller linked to immunity enzyme

An enzyme implicated in innate immunity offers a potential new target for treating liver injury due to acetaminophen, a common painkiller also known as paracetamol in many countries. A team from South Korea led by Seok Hee Park from Sungkyunkwan University in Suwon and Seong-Jin Kim from the GILO Foundation in Seoul showed that an enzyme called Pellino3 plays a critical role in triggering liver damage caused by acetaminophen. The researchers found that mice lacking Pellino3, an enzyme that marks proteins with chemical tags to mediate immune responses, showed reduced liver damage as measured by several indicators of liver stress. Pellino3 usually activates a specific pro-inflammatory protein and drugs directed at such activation could help protect the liver in regular users of this analgesic and anti-fever drug.

## Introduction

Acetaminophen (*N*-acetyl-*p*-aminophenol; APAP) is a mild analgesic and antipyretic drug commonly used worldwide. However, APAP overdose can cause severe liver damage that progresses to acute liver failure and death, even though APAP is recognized to be safe at proper therapeutic doses^1^. A large body of evidence accumulated over the past decades points to the accumulation of *N*-acetyl-*p*-benzoquinone imine (NAPQI), the reactive and toxic metabolite of APAP, as the main cause of liver injury induced by APAP overdose^2^. Although small amounts of NAPQI, which is metabolized from APAP by hepatic cytochrome P450 2E1 (CYP2E1), can be detoxified by glutathione (GSH) conjugation, excessive formation of NAPQI after APAP overdose rapidly depletes hepatic GSH and thus causes the formation of protein adducts, particularly in mitochondrial proteins^3,4^. This mitochondrial protein adduct formation induces excessive generation of mitochondrial superoxide, which can react with nitric oxide (NO) to produce peroxynitrite, and thereby interferes with protein function through nitration of protein tyrosine residues^5,6^. Protection against APAP-induced hepatoxicity by the superoxide dismutase (SOD) mimetic Mito-TEMPO, together with increased injury in partial SOD-deficient mice, emphasizes the importance of superoxide in this process^7–9^. Furthermore, several studies of APAP-induced liver injury indicate that neuronal nitric oxide synthase (nNOS) is involved in the elevation of NO^10–12^. These mitochondrial changes in APAP hepatotoxicity, such as severe oxidative stress and nitrosative stress, contribute to the opening of mitochondrial permeability transition pores, which leads to loss of the mitochondrial membrane potential and the release of endonucleases that cause DNA damage^13^. All these events ultimately result in hepatocellular necrosis, although recent studies have also suggested the importance of necroptosis^14–18^. Necrotic cell death in APAP hepatotoxicity releases damage-associated molecular patterns (DAMPs), including mitochondrial DNA, nuclear DNA fragments, High-mobility group box 1 (HMGB1) protein and ATP, which results in sterile inflammation^19,20^. These DAMPs activate inflammasome signaling by binding to pattern recognition receptors (PRRs) and promote the processing of pro-caspase-1, which results in the subsequent processing of pro-IL-1β or pro-IL-18 into active cytokines^21^. These cytokines appear to be responsible for the activation and recruitment of neutrophils and monocyte-derived macrophages into the liver, although whether these inflammatory cells aggravate liver injury or facilitate regeneration remains controversial^19^. In addition to these processes, it has been reported that autophagy plays an important role in facilitating liver recovery and regeneration^22–24^.

Despite extensive pathophysiological studies on liver injury and the reactive metabolites generated by APAP overdose, the signaling pathways governing this hepatotoxicity are likely to be more complicated than our current knowledge. Considerable studies have mainly demonstrated a detrimental role of c-Jun N-terminal kinase (JNK), a serine/threonine protein kinase that belongs to the mitogen-activated protein kinase (MAPK) superfamily, in APAP-induced hepatotoxicity^25–30^. This sustained activation of JNK in the cytosol and its mitochondrial translocation during APAP overdose are believed to amplify APAP-mediated mitochondrial and nitrosative stress^31^.

In APAP-mediated hepatotoxicity, JNK activation has been reported to be modulated by upstream signaling proteins such as GSK3β, MLK3, and ASK1^32–34^. However, recent reports also indicate that protein kinase C (PKC) and receptor-interacting protein kinase 1 (RIPK1) participate in APAP hepatotoxicity through JNK activation^35,36^. Among these kinases, GSK3β is known to be an important mediator involved in APAP-mediated hepatotoxicity through its translocation into mitochondria as well as JNK activation^32^.

Despite the current knowledge regarding the signaling pathways involved in APAP hepatotoxicity, the ubiquitin-modifying systems in APAP-mediated liver injury is less understood than the phosphorylation of signaling components. The ubiquitination of target proteins has been found to be crucial for diverse cellular processes such as protein stability, signal transduction, endocytosis and trafficking, and this versatile modification is mainly mediated by E3 ubiquitin ligases^37^. The Pellino3 protein, encoded by the *Peli3* gene, is a member of the Pellino family composed of Pellino1, Pellino2 and Pellino3^38^. Pellino3 has a RING domain with E3 ligase activity and is involved in diverse inflammatory signaling pathways, such as Toll-like receptor signaling, NOD2 signaling, and TNF signaling pathways^39–42^. Interestingly, overexpression of Pellino3 proteins has been found to facilitate the activation of MAPKs such as JNK and p38^43,44^. These results strongly suggest a possible role of Pellino3 in APAP-mediated hepatotoxicity, which has not been addressed until now.

In this study, we demonstrate a role of the Pellino3 protein in APAP-mediated hepatotoxicity through its involvement in the mitochondrial translocation and phosphorylation of GSK3β through the use of whole-body *Peli3*^−/−^ knockout (KO) and adenovirus-mediated *Peli3* knockdown (KD) mouse models. This report provides the first identification of an E3 ubiquitin ligase modulating the function of GSK3β in APAP-mediated liver injury.

## Materials and methods

### Generation of *Peli3*^−/−^ KO mice

To generate *Peli3*^−/−^ KO mice, a conditional knockout vector targeting the *Peli3* gene was construed using a recombineering system^45^ (Supplementary Fig. 1a). A 5.9 kb genomic DNA fragment containing *Peli3* exon 2 was inserted into the pLMJ235 plasmid. The loxP sequence and frt-loxP-Neo-frt-loxP cassette, which has a positive selection marker (neomycin resistance gene), were inserted 117 bp upstream and 304 bp downstream of exon 2, respectively. The linearized targeting vector against the *Peli3* gene was initially electroporated into 2 × 10^7^ J1 mouse ESCs. Approximately three hundred G418- and 1-(2-deoxy-2-fluoro-1-β-D-arabinofuranosyl)-5-iodouracil (FIAU)-resistant colonies were randomly picked, and these clones were screened by genomic Southern blot analyses using external probes. Targeted ESCs were injected into blastocysts of the C57BL/6 (B6) strain. ESC culture and blastocyst injections were performed using standard methods. Male chimeras were bred with B6 females to establish the *Peli3*^+/3f^ strain in a 129/B6 hybrid background. *Peli3*^+/3f^ mice were crossed with β-actin-Cre mice to generate a mouse strain with the *Peli3*^1*f*^ null allele^46^, in which exon 2 was deleted. To generate *Peli3* KO mice with the C57BL/6 background, *Peli3* KO mice with the hybrid background were backcrossed with C57BL/6 mice for more than ten generations. Genotypes of wild-type and *Peli3*^−/−^ KO mice were analyzed by PCR using the indicated primers (Supplementary Fig. 1b). The expression of *Peli3* mRNA in *Peli3*^−/−^ KO mice was also confirmed by quantitative real-time RT‒PCR (qRT‒PCR) with the primers described in Supplementary Table 1.

### Cell culture, reagents, and transfection

Human embryonic kidney 293 (HEK293) cells were purchased from American Type Culture Collection (Manassas, VA, USA). HEK293 cells were maintained in Dulbecco’s modified Eagle’s medium (DMEM; Thermo Fisher Scientific, Waltham, MA, USA) with 10% fetal bovine serum (FBS; Thermo Fisher Scientific), 10 units/ml penicillin and 10 mg/ml streptomycin (Thermo Fisher Scientific). The HEK293 cells were routinely tested for mycoplasma contamination by PCR. Mouse primary hepatocytes were incubated in M199 medium (Sigma-Aldrich) with 10% FBS, 10 units/ml penicillin, 10 mg/ml streptomycin, 23 mM HEPES, and 10 nM dexamethasone (Sigma-Aldrich). Acetaminophen (APAP) was purchased from Sigma-Aldrich. Plasmids were transiently transfected into HEK293 cells using polyethylenimine (PEI). Lipofectamine 2000 (Thermo Fisher Scientific) was used for transfection into mouse primary hepatocyte cells.

### Animal experiments

Male *Peli3*^−/−^ KO mice (8–9 weeks of age) and their wild-type littermates were used for the overall experiments, were housed at a pathogen-free facility, maintained a 12 h/12 h circadian clock rhythm and were fed standard chow. To establish an APAP-induced liver injury model, mice that had fasted for 14 h in the presence of water without only food were orally administered 500 mg/kg APAP. Prior to its administration, APAP was dissolved in distilled water at 55 °C and cooled to 37 °C. As a control, distilled water was orally administered to the mice. After the administration of APAP, the survival of the mice was monitored at the indicated times. Liver tissue and blood serum were collected 24 h after APAP treatment for further experiments. The construction of recombinant adenoviruses expressing a nonspecific RNAi control (shCON) and *Peli3*-specific shRNAs (shPeli3 #3 and shPeli3 #4) and the related animal experiments were performed as previously described^47^. The specific RNAi sequences against endogenous *Peli3* mRNA used in the recombinant adenoviruses are described in Supplementary Table 2. This study was reviewed and approved by the Institutional Animal Care and Use Committee (IACUC) of Sungkyunkwan University School of Medicine (SUSM), which is an Association for Assessment and Accreditation of Laboratory Animal Care International (AAALAC International) accredited facility and abides by the Institute of Laboratory Animal Resources (ILAR) guidelines. In all animal experiments, mice were randomly selected for generation of the APAP-induced liver injury model.

### Plasmids

HA-tagged human Pellino3a and Pellino3b expression plasmids were previously described^48^. Using HA-hPellino3a and HA-hPellino3b as templates for PCR with specific primers, Pellino3a and Pellino3b cDNAs were subcloned into the *Eco*RI and *Xho*I sites of the pcDNA-Flag vector (Thermo Fisher Scientific) or the *Xho*I and *Kpn*I sites of the pCS3+MTBX vector, which was kindly provided by Dr. C.Y. Choi (Sungkyunkwan University, Korea), and this process yielded Flag-hPellino3a and Flag-hPellino3b and 6xMyc-hPellino3a and 6xMyc-hPellino3b, respectively. Full-length mouse Pellino3 cDNA was amplified from mouse primary hepatocytes and subcloned into the *Eco*RI and *Xho*I sites of the pcDNA-Flag vector or the *Xho*I and *Sal*I sites of the pCS3+MTBX vector, resulting in Flag-mPellino3 or Myc-mPellino3. Full-length human GSK3β cDNA was amplified by PCR from a previously described plasmid encoding HA-GSK3β^49^ and then subcloned into the *Eco*RI and *Sal*I sites of the pCS3+MTBX vector or the *Eco*RI and *Nco*I sites of the pCS5-Flag vector to yield 6xMyc-GSK3β or Flag-GSK3β. Full-length mouse GSK3β was subcloned into the *Eco*RV and *Nco*I sites of the pCS5-Flag vector, resulting in Flag-mGSK3β. Full-length JNK1, JNK2 and MLK3 cDNAs were purchased from Addgene (Watertown, MA, USA) and subcloned into the *Eco*RV and *Xho*I sites of the pcDNA-Flag vector after PCR amplification. Full-length human MKK4 and MKK7 cDNAs were amplified from the cDNAs of HEK293 cells and subcloned into the *Bam*HI/*Xho*I and *Eco*RI/*Xho*I sites of the pcDNA-Flag vector, which yielded Flag-MKK4 and Flag-MKK7, respectively. Catalytically inactive mutants of human Pellino3a and Pellion3b and mouse Pellino3 cDNAs were generated using the QuikChange Mutagenesis kit (Stratagene, La Jolla, CA, USA) using Flag-hPellino3a, Flag-hPellino3b, and Flag-mPellino3 as templates, which yielded Flag-hPellino3a-CI, Flag-hPellino3b-CI, and Flag-mPellino3-CI. The mouse GSK3β-S9A mutant was also generated using the QuikChange Mutagenesis kit, resulting in Flag-mGSK3β-S9A. Plasmids encoding HA-tagged ubiquitin (HA-Ubi) and His-tagged ubiquitin (His-Ubi) were previously described^47,48^. PCR-generated portions of all constructs in this study were verified by sequencing. The sequences of the primers used for PCR amplification and site-directed mutagenesis in this study are described in Supplementary Table 3.

### Antibodies, RNA extraction and quantitative real-time RT‒PCR

Immunoblot and immunoprecipitation assays were performed as previously described^49^. The company names, catalog numbers, species, and dilution ratios of the antibodies used for immunoblotting and immunoprecipitation are described in Supplementary Table 4. The isolation of total RNA and cDNA synthesis were performed as previously described^49^. The sequences of the primers for *Peli3*, *Tnf* and *Ifn1* mRNAs used for real-time qRT‒PCR are described in Supplementary Table 1. Real-time qRT‒PCR was performed using a CFX Connect real-time PCR machine and iQ SYBR Green SuperMix (Bio-Rad, Hercules, CA, USA) to measure the expression of genes under the following conditions: 40 cycles of 95 °C for 10 s, 62 °C for 10 s, and 72 °C for 30 s. All reactions were independently repeated at least three times to ensure reproducibility.

### Statistical analysis

All experiments, including immunoblots, were performed with three independent biological replicates. The results are expressed as the means ± SDs. Statistical significance was calculated by one-way or two-way ANOVA using GraphPad Prism 5 software (GraphPad, La Jolla, CA, USA). *P* < 0.05 was considered to indicate statistical significance.

Details of the histological analysis, neutrophil infiltration, terminal deoxynucleotidyl transferase-mediated deoxyuridine triphosphate nick-end labeling (TUNEL) assay, primary hepatocyte isolation, glutathione and reactive oxygen species measurements, 3-(4,5-dimethylthiazol-2-yl)-2,5-diphenyltetrazolium bromide (MTT) and lysosomal activity assays, fractionation of cytosolic and mitochondrial extracts, immunoblotting, immunoprecipitation, in vitro ubiquitination assay, ELISA and myeloperoxidase (MPO) assays are provided in the Supplementary Information.

## Results

### The ablation of *Peli3* in hepatocytes decreases APAP-induced liver injury

**Fig. 1:**
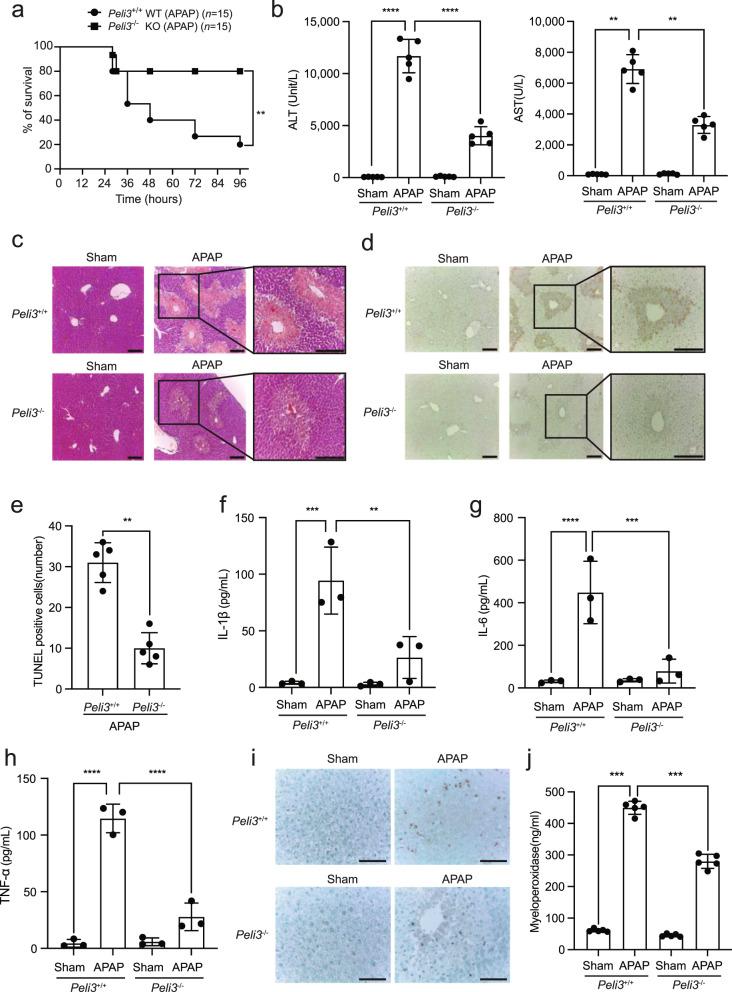
Ablation of the *Peli3* gene diminishes APAP-induced liver injury. **a** *Peli3*^−/−^ mice and wild-type (WT) littermates were orally administered 500 mg/kg APAP, and the overall survival of the mice was monitored at the indicated times. *n* = 15 per group. The data were statistically analyzed by the log-rank test (^**^P < 0.05 compared to the control group, *Peli3*^+/+^ WT mice). **b** The levels of serum ALT and AST were measured at 24 h after the oral administration of APAP. *n* = 5 per group. Sham indicates the administration of distilled water (DW). The data were statistically analyzed by two-way ANOVA followed by Bonferroni’s multiple comparison test (^**^P < 0.01 and ^****^P < 0.0001 compared to the indicated group). The bars represent the means ± SDs. **c** H&E staining of livers isolated from *Peli3*^−/−^ and WT mice at 24 h after the oral administration of APAP. Scale bars, 100 μM (original images), 500 μm (magnified insets). **d** TUNEL assays of the livers of *Peli3*^−/−^ and WT mice at 24 h after oral administration. Scale bars, 100 μm (original images), 500 μm (magnified insets). **e** At least five hot spots in a section from the TUNEL assays were selected, and the average count was determined. The data were statistically analyzed by two-way ANOVA followed by Sidak’s multiple comparison test (^**^P < 0.01 compared to the indicated group). The bars represent the means ± SDs. **f**–**h** ELISAs of pro-inflammatory cytokines in the sera of *Peli3*^−/−^ KO and *Peli3*^+/+^ WT mice at 24 h after the oral administration of APAP. *n* = 3 per group. The data were statistically analyzed by two-way ANOVA followed by Bonferroni’s multiple comparison test (^**^P < 0.01, ^***^P < 0.001, and ^****^P < 0.0001 compared to the indicated group). **i** Immunohistochemistry for the neutrophil markers Ly6G/Ly6C in liver tissue sections of *Peli3*^−/−^ and WT mice at 24 h after the oral administration of APAP. Scale bars, 100 μm. **j** Myeloperoxidase activities in liver lysates obtained from *Peli3*^−/−^ and WT mice at 24 h after the oral administration of APAP. *n* = 5 per group. The data were statistically analyzed by two-way ANOVA followed by Bonferroni’s multiple comparison test (^***^P < 0.001 compared to the indicated group). The bars represent the means ± SDs. In (**c**), (**d**) and (**i**), the images shown are representative of three independent experiments.

Although the Pellino3 protein, with its intrinsic E3 ligase activity, has been reported to be involved in the activation of MAPKs, such as JNK and p38^43,44^, its pathophysiological role in APAP-induced liver injury is unknown. To address the exact function of Pellino3 in APAP-induced liver injury, we generated whole-body *Peli3*^−/−^ KO mice (Supplementary Fig. 1a). The knockout of the *Peli3* gene was confirmed by genotyping and quantitative real-time RT‒PCR because all commercially available antibodies against the Pellino3 protein could not detect endogenous Pellino3 expression (Supplementary Fig. 1b, c). Whole-body *Peli3*^−/−^ KO mice and wild-type (*Peli3*^+/+^ WT) mice were orally administered a high dose of APAP (500 mg/kg), and their survival rates were observed for 4 days. At Day 4, *Peli3*^−/−^ KO mice (*n* = 15) showed an 80% survival rate, whereas *Peli3*^+/+^ WT mice (*n* = 15) showed a 20% survival rate (*P* < 0.05) (Fig. 1a). Furthermore, the levels of biomarkers of liver injury, namely, serum alanine aminotransferase (ALT) and aspartate aminotransferase (AST), were significantly decreased in *Peli3*^−/−^ KO mice compared to *Peli3*^+/+^ WT mice (Fig. 1b). Consistent with these results, hematoxylin and eosin (H&E) staining of liver tissues indicated that centrilobular necrotic cell death and immune cell infiltration, which are induced by APAP treatment in *Peli3*^+/+^ WT mice, were significantly reduced in *Peli3*^−/−^ KO mice (Fig. 1c). The TUNEL assay of *Peli3*^−/−^ KO mice supported the reduction in cell death in the liver (Fig. 1d, e). In addition, the levels of pro-inflammatory cytokines such as IL-1β, IL-6, and TNFα were significantly reduced upon APAP treatment in the sera of *Peli3*^−/−^ KO mice compared to *Peli3*^+/+^ WT mice (Fig. 1f–h). Consistent with the decreased expression of these cytokines, immunohistochemistry for the neutrophil markers Ly6G and Ly6C and MPO assays showed reduced infiltration of neutrophils in the livers of *Peli3*^−/−^ KO mice (Fig. 1i, j).

### The hepatocyte-specific depletion of *Peli3* mRNA decreases APAP-induced liver injury

**Fig. 2:**
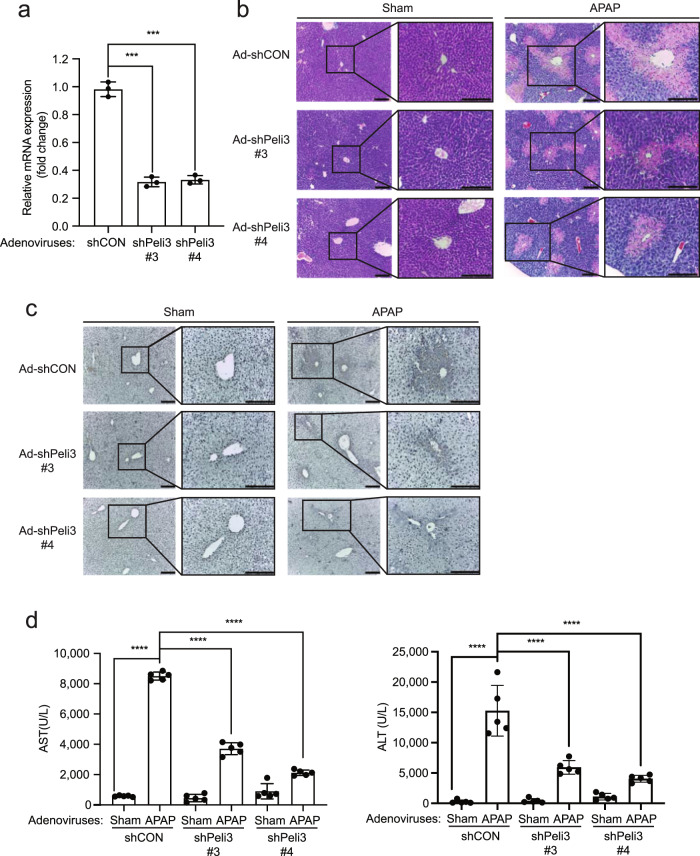
The hepatocyte-specific knockdown of the *Peli3* gene decreases APAP-induced liver injury. **a** Mice were infected with adenoviruses expressing shRNA targeting *Peli3* mRNA (Ad-shPeli3 #3 and Ad-shPeli3 #4) through the tail vein. Adenoviruses expressing nonspecific shRNA (Ad-shCON) were used as a control. The knockdown of *Peli3* mRNA in hepatocytes at 96 h post-infection of these viruses was confirmed by qRT‒PCR analysis. *n* = 3 per group. The data were statistically analyzed by two-way ANOVA followed by Sidak’s multiple comparison test (^***^P < 0.001 compared to the indicated group). The bars represent the means ± SDs. **b,** **c** Mice were infected with recombinant adenoviruses, and 72 h later, the mice were fasted for 14 h and then orally administered 500 mg/kg APAP. H&E staining (**b**) and TUNEL assays (**c**) of livers isolated from *Peli3* knockdown (Ad-shPeli3 #3 and Ad-shPeli3 #4) and control mice (Ad-shCON) at 24 h after the oral administration of APAP were performed. Scale bars, 100 μm (original images) and 500 μm (magnified insets). The images are representative of three independent experiments. **d** The levels of serum ALT and AST were measured at 24 h after the oral administration of APAP. *n* = 5 per group. The data were statistically analyzed by two-way ANOVA followed by Bonferroni’s multiple comparison test (^**^P < 0.01, ^***^P < 0.001, and ^****^P < 0.0001 compared to the indicated group). The bars represent the means ± SDs. **b**–**d** Sham indicates the administration of distilled water (DW).

To demonstrate a hepatocyte-specific role of the *Peli3* gene in APAP-induced liver injury, we generated mice with acute liver-specific depletion of *Peli3* mRNA by injecting two adenoviruses expressing different shRNAs against *Peli3* mRNA (Ad-shPeli3 #3 and Ad-shPeli3 #4) via the tail vein. As a negative control, we used an adenovirus expressing nonspecific shRNA (Ad-shCON). Quantitative real-time RT‒PCR (qRT‒PCR) analysis showed significant downregulation of *Peli3* mRNA in primary hepatocytes expressing recombinant adenoviruses with each shRNA against *Peli3* mRNA compared to the control adenoviruses (Fig. 2a). Nice were infected with these adenoviruses for 72 h and subsequently fasted for 14 h, and 500 mg/kg APAP was then orally administered. H&E staining at 24 h post APAP treatment indicated that liver damage induced by APAP treatment, such as centrilobular necrotic cell death, was significantly decreased in the livers of *Peli3* KD mice (Ad-shPeli3 #3 and #4) (Fig. 2b). TUNEL assays also indicated a reduction in cell death in the livers of *Peli3* KD mice (Fig. 2c). Furthermore, the serum ALT and AST levels were significantly decreased in *Peli3* KD mice compared to control mice (Fig. 2d). These results strongly suggest that *Peli3* deficiency in hepatocytes decreases APAP-induced liver injury.

Since Pellino3 has been shown to be required for the regulation of diverse inflammatory signaling pathways, such as Toll-like receptor signaling, NOD2 signaling, and TNF signaling pathways^39–42^, we next examined the expression of target genes such as TNFα and type I interferon in hepatocytes to understand whether Pellino3 mediates APAP hepatotoxicity through regulation of the same target genes. Although *Peli3* deficiency inhibits expression of TNFα induced by the NOD2 ligand in bone marrow-derived macrophages and augments the expression of IFNβ induced by poly(I:C)^39,41^, *Peli3* deficiency in hepatocytes did not result in significant differences in the expression of TNFα and IFNβ mRNAs in the context of APAP treatment (Supplementary Fig. 2a, b). In contrast, qRT‒PCR analysis of whole liver extracts indicated that the expression levels of both TNFα and IFNβ upon APAP treatment were decreased in *Peli3*^−/−^ KO mice compared to *Peli3*^+/+^ WT mice (Supplementary Fig. 2c, d). This discrepancy appears to be due to the characteristics of whole liver extracts that include immune cells and hepatocytes. Therefore, it is possible that the mechanistic role of Pellino3 in APAP hepatotoxicity may be distinct from its role in inflammatory signaling pathways. However, it is noteworthy that the IFNβ expression levels in the context of APAP treatment are opposite to a previous finding regarding in NOD2 signaling^39^, which suggests the possibility that Pellino3 contributes to APAP hepatotoxicity through a mechanism that has not been identified based on the current knowledge regarding Pellino3.

### *Peli3* depletion has therapeutic effects on APAP hepatotoxicity

**Fig. 3:**
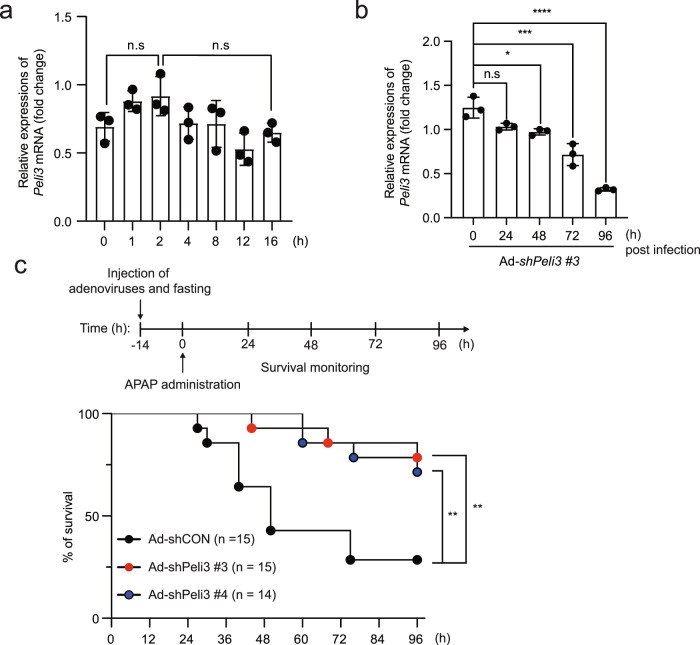
*Peli3* knockdown after APAP treatment increases the survival rate. **a** The expression of *Peli3* mRNA in normal primary hepatocytes at the indicated time points after APAP treatment was confirmed by qRT‒PCR analysis. **b** The reduction of *Peli3* mRNA expression in primary hepatocytes infected with recombinant adenoviruses expressing *Peli3*-specific shRNA (Ad-shPeli3 #3) at the indicated times was confirmed by qRT‒PCR analysis. **a**, **b** The data are representative of three independent experiments and statistically analyzed by two-way ANOVA followed by Sidak’s multiple comparison test (^***^P < 0.001 compared to the indicated group). n.s; not significant. The bars represent the means ± SDs. **c** *Peli3*^+/+^ WT mice were infected with recombinant adenoviruses expressing *Peli*3-specific shRNAs (Ad-shPeli3 #3 and Ad-shPeli3 #4) or control adenoviruses expressing nonspecific shRNA (Ad-shCON) and were subsequently fasted for 14 h. APAP (500 mg/kg) was orally administered, and the overall survival of the mice was monitored at the indicated times. *n* = 14 or 15 per group. The data were statistically analyzed by the log-rank test (^**^P < 0.05 compared to the control group, Ad-shCON).

We next investigated whether *Peli3* depletion after the onset of APAP overdose shows therapeutic effects. To this end, we first examined the expression levels of *Peli3* mRNA in primary hepatocytes during APAP treatment. Real-time qRT‒PCR analysis indicated that *Peli3* expression in hepatocytes was minimally affected by APAP treatment at early and late time points (Fig. 3a). To determine the time points at which recombinant adenovirus expressing shRNA against *Peli3* mRNA reduces the endogenous *Peli3* mRNA levels, *Peli3*^+/+^ WT mice were injected with recombinant adenoviruses (Ad-shPeli3 #3) via the tail vein. After isolation of primary hepatocytes at the indicated time points, the expression of *Peli3* mRNA was analyzed by qRT‒PCR. Although endogenous *Peli3* mRNA expression was not affected at 24 h after injection of Ad-shPeli3 #3 viruses, a significant reduction was initially detected at 72 h after injection (Fig. 3b). Based on these results, WT mice were infected with recombinant adenoviruses, fasted for 14 h, and orally administered 500 mg/kg APAP, and their survival was subsequently observed (Fig. 3c). *Peli3* depletion by recombinant adenoviruses significantly increased the survival rates at both 72 h and 96 h after the onset of APAP overdose compared with those of WT mice injected with control adenoviruses (Ad-shCON) (Fig. 3c). These results suggest that *Peli3* KD may have therapeutic potential in the treatment of APAP-induced liver injury.

### *Peli3* deficiency reduces APAP-induced oxidative stress

**Fig. 4:**
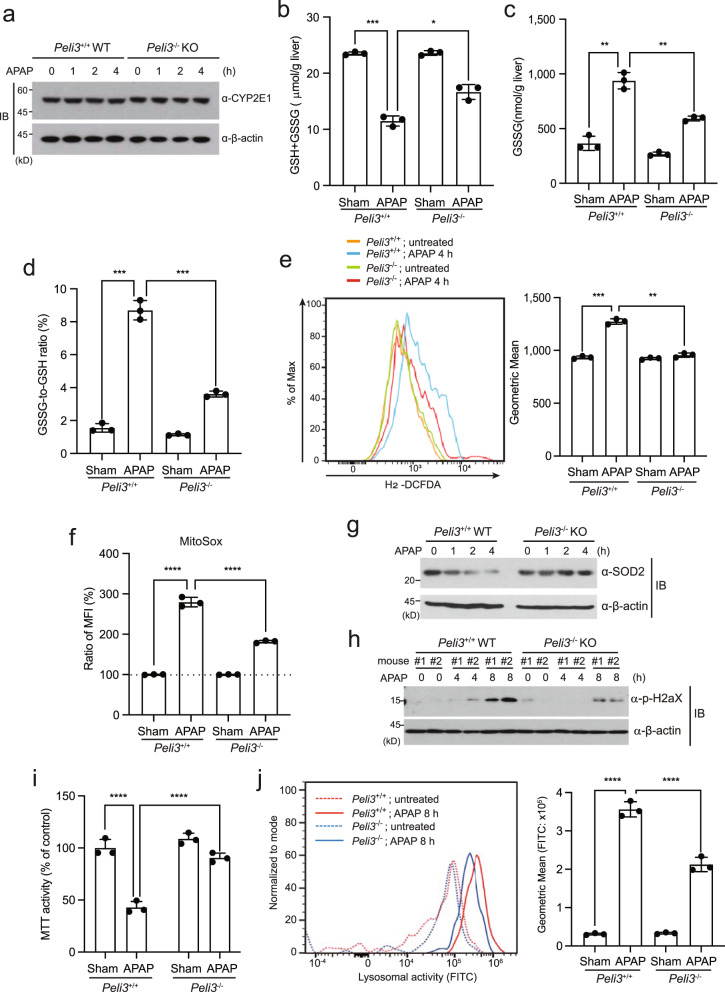
*Peli3* deficiency suppresses ROS generation as well as mitochondrial and lysosomal damage. **a** The expression of hepatic cytochrome P450 2E1 (*CYP2E1*) protein in the primary hepatocytes of *Peli3*^−/−^ KO and *Peli3*^+/+^ WT mice was monitored during treatment with 20 mM APAP for the indicated times. **b**–**d** The levels of total glutathione (**b**: GSH + GSSG) and oxidized glutathione (**c**: GSSG) and the GSSG-to-GSH ratio (**d**) in the primary hepatocytes of *Peli*3^−/−^ and WT mice were measured at 2 h post 20 mM APAP treatment. *n* = 3 per group. **e** Reactive oxygen species (ROS) in primary hepatocytes were measured by flow cytometry using H_2_-DCFDA and quantified. *n* = 3 per group. **f** Primary hepatocytes of *Peli3*^−/−^ KO and *Peli3*^+/+^ WT mice were incubated with MitoSOX Red dye, treated with 20 mM APAP for 4 h, and analyzed by flow cytometry for MitoSOX fluorescence. *n* = 3 per group. **g** The expression of superoxide dismutase 2 (SOD2) in the primary hepatocytes of *Peli3*^−/−^ KO and *Peli3*^+/+^ WT mice was monitored during treatment with 20 mM APAP for the indicated times. **h** The phosphorylation of histone H2AX in primary hepatocytes obtained from two independent *Peli3*^−/−^ KO and *Peli3*^+/+^ WT mice was monitored by immunoblotting at the indicated times. (**i**) For the evaluation of mitochondrial function, MTT assays of primary hepatocytes of *Peli3*^−/−^ KO and *Peli3*^+/+^ WT mice collected after treatment with APAP for 4 h were performed. *n* = 3 per group. **j** A lysosomal activity assay using a self-quenched substrate was performed with primary hepatocytes of *Peli3*^−/−^ KO and *Peli3*^+/+^ WT mice after treatment with APAP for 8 h. *n* = 3 per group. **b**–**f**, **i**, **j** The data were statistically analyzed by two-way ANOVA followed by Bonferroni’s multiple comparison test (^*^P < 0.05, ^**^P < 0.01, ^***^P < 0.001, and ^****^P < 0.0001 compared to the indicated group). The bars represent the means ± SDs. The images shown in (**a**), (**g**) and (**h**) are representative of three independent experiments.

Because *Peli3* deficiency decreases APAP-induced liver injury, it is possible that Pellino3 affects APAP metabolism. To confirm this possibility, we investigated the expression of the *CYPE21* gene, which encodes hepatic cytochrome P450 2E1 metabolizing APAP. Immunoblot analysis indicated that the expression of the *CYP2E1* gene in *Peli3*^−/−^ KO hepatocytes was similar to that in *Peli3*^+/+^ WT hepatocytes, indicating that *Peli3* deficiency does not affect APAP metabolism (Fig. 4a). We next examined the GSH levels in the livers of *Peli3*^−/−^ KO mice compared to *Peli3*^+/+^ WT mice because GSH depletion is a major component of APAP-induced hepatotoxicity^2^. To this end, we isolated primary hepatocytes from *Peli3*^+/+^ WT and *Peli3*^−/−^ KO mice and subsequently treated them with 20 mM APAP for 2 h. As expected, the total GSH levels, which included both oxidized glutathione (GSSG) and reduced glutathione (GSH), were decreased in the primary hepatocytes of *Peli3*^+/+^ WT mice upon APAP treatment (Fig. 4b). However, then total GSH levels in *Peli3*^−/−^ KO mice were not reduced as much as those in *Peli3*^+/+^ WT mice (Fig. 4b). Consistent with these results, the oxidized glutathione (GSSG) levels and the ratio of GSSG to GSH were increased in the primary hepatocytes of *Peli3*^+/+^ WT mice and significantly decreased in *Peli3*^−/−^ KO mice (Fig. 4c, d). These results indicated that *Peli3* deficiency leads to a reduction in GSH depletion in APAP-induced liver injury.

Because GSH depletion in APAP hepatotoxicity is related to the generation of ROS, we next measured the ROS levels in the primary hepatocytes of *Peli3*^+/+^ WT and *Peli3*^−/−^ KO mice upon 20 mM APAP treatment by flow cytometry analysis using H_2_-DCFDA dye. At 4 h post APAP treatment, the ROS levels were increased in the hepatocytes of *Peli3*^+/+^ WT mice and significantly decreased in those of *Peli3*^−/−^ KO mice (Fig. 4e). In addition to the intracellular ROS, MitoSOX staining indicated that the levels of mitochondrial ROS were significantly reduced upon APAP treatment in *Peli3*^−/−^ KO hepatocytes compared to *Peli3*^+/+^ WT hepatocytes (Fig. 4f). Furthermore, the expression of mitochondrial superoxide dismutase 2 (SOD2), which acts as a scavenger protein to eliminate ROS, was sustained in APAP-treated primary hepatocytes of *Peli3*^−/−^ KO mice, whereas reduced expression was observed in *Peli3*^+/+^ WT mice (Fig. 4g). Since oxidative and nitrosative stress in APAP hepatotoxicity have been known to induce DNA damage and mitochondrial dysfunction^50^, we next examined the phosphorylation of histone H2AX (pH2AX), a hallmark of double-strand breaks (DSBs), in hepatocytes obtained from *Peli3*^+/+^ WT and *Peli3*^−/−^ KO mice upon APAP treatment. The phosphorylation of histone H2AX was significantly decreased in *Peli3*^−/−^ KO mice (Fig. 4h). To evaluate mitochondrial function in *Peli3*^−/−^ KO mice, we assessed mitochondrial dehydrogenase activity by the MTT assay. Mitochondrial function in *Peli3*^+/+^ WT hepatocytes, but not in *Peli3*^−/−^ KO hepatocytes, was significantly impaired after APAP treatment (Fig. 4i). Next, using a self-quenched substrate, we quantified the in situ lysosomal enzyme activity in the primary hepatocytes of *Peli3*^−/−^ KO and *Peli3*^+/+^ WT mice after treatment with APAP for 8 h. Lysosomal enzyme activity was increased by APAP in *Peli3*^+/+^ hepatocytes, reflecting the lysosomal damage caused after APAP treatment, whereas the enzyme activity was reduced in *Peli3*^−/−^ hepatocytes (Fig. 4j). These results provide robust evidence showing that *Peli3* deficiency contributes to the suppression of mitochondrial and lysosomal damage by decreasing oxidative stress upon APAP treatment.

### Pellino3 induces K63-linked polyubiquitination of GSK3β in APAP-induced liver injury

Next, we explored the molecular function of the Pellino3 protein in APAP-induced liver injury. To find a clue, we first examined the interaction of the Pellino3 protein with diverse kinases involved in APAP-induced liver injury because Pellino3 activity has been shown to lead to the activation of JNK^43^. There are two forms of Pellino3 proteins in humans produced by alternative splicing, Pellino3a and Pellino3b^43^, whereas a single form of Pellino3 is found in mice. The human Pellino3 and mouse Pellino3 proteins used in this study are designated hPellino3 and mPellino3, respectively.

**Fig. 5:**
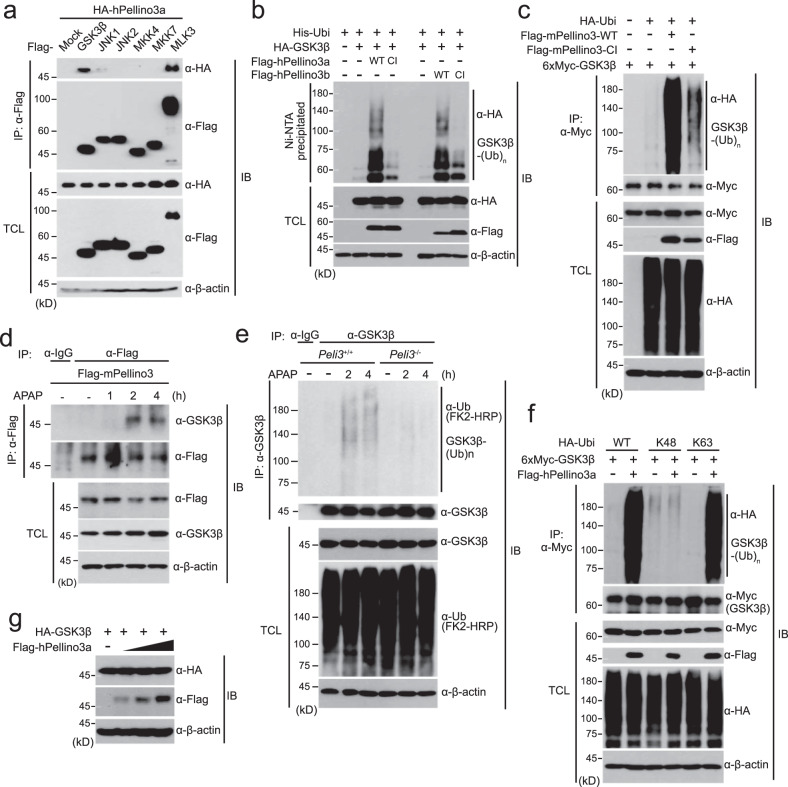
Pellino3 induces the K63-linked polyubiquitination of GSK3β through direct binding. **a** A plasmid encoding HA-hPellino3 was cotransfected into HEK293 cells with the indicated Flag-tagged plasmids. Cell lysates were immunoprecipitated (IP) with anti-Flag antibody and subsequently immunoblotted (IB) with anti-HA and anti-Flag antibodies. Total cell lysates (TCLs) were immunoblotted with the indicated antibodies. **b** After a plasmid encoding wild-type His-Ubi was cotransfected with HA-GSK3β into HEK293 cells in the absence or presence of Flag-hPellino3a, Flag-hPellino3a-CI, Flag-hPellin3b and Flag-hPellino3b-CI, Ni-NTA-mediated pull-down assays were performed. TCLs were immunoblotted with the indicated antibodies. **c** Plasmids encoding 6xMyc-GSK3β and HA-Ubi were cotransfected into HEK293 cells together with a plasmid encoding wild-type Flag-mPellino3 or a catalytically inactive (CI) mutant of Flag-mPellino3 (Flag-mPellino3-CI). GSK3β ubiquitination was examined by immunoprecipitation using an anti-Myc antibody under 1% SDS denaturing conditions and immunoblotting with an anti-HA antibody. TCLs were immunoblotted with the indicated antibodies. **d** A plasmid encoding Flag-mPellino3 was transfected into *Peli3*^−/−^ KO hepatocytes and subsequently treated with 20 mM for the indicated times. Cell lysates were immunoprecipitated with anti-Flag antibody and subsequently immunoblotted with anti-GSK3β antibody. TCLs were immunoblotted with the indicated antibodies. **e** For the ubiquitination of endogenous GSK3β protein, primary hepatocytes of *Peli3*^−/−^ and WT mice were treated with 20 mM APAP for 2 h or 4 h. Cell lysates were immunoprecipitated with anti-GSK3β antibody under 1% SDS denaturing conditions and subsequently immunoblotted with anti-ubiquitin (FK2-HRP) antibody. TCLs were immunoblotted with the indicated antibodies. As a negative control, cell lysates were immunoprecipitated with anti-IgG antibody. **f** A plasmid encoding wild-type ubiquitin (HA-Ubi-WT) or ubiquitin mutants that only mediate K48-linked (HA-Ubi-K48) or K63-linked (HA-Ubi-K63) polyubiquitination was transfected into HEK293 cells with 6xMyc-GSK3β and Flag-hPellino3a at the indicated combinations. Cell lysates were immunoprecipitated (IP) with anti-Myc antibody and subsequently immunoblotted (IB) with anti-HA antibody. TCLs were immunoblotted with the indicated antibodies. **g** A plasmid encoding HA-GSK3β was cotransfected into HEK293 cells together with dose-dependent increased expression of Flag-hPellino3a. Cell lysates were immunoblotted with the indicated antibodies. In all immunoblots, the expression of β-actin was used as a loading control. The images in this figure are representative of at least three independent experiments.

Coimmunoprecipitation assays revealed that the human Pellino3a protein, which is the longer form, strongly bound to GSK3β and MLK3, which are MAP3Ks, but bound weakly to JNKs (Fig. 5a). Similarly, hPellino3b, the shorter form, also bound to GSK3β and MLK3 (Supplementary Fig. 3a, b). These results prompted us to investigate whether the human Pellino3 protein with its E3 ligase activity induces the ubiquitination of GSK3β and MLK3. Plasmids encoding HA-GSK3β and His-Ubi were cotransfected into HEK293 cells in the presence of wild-type Flag-hPellino3, Flag-hPellino3b, or catalytically inactive mutants in which cysteine residues within the active site of hPellino3a or hPellino3b were mutated into alanines, and Ni-NTA pull-down assays were then performed. The polyubiquitination of GSK3β was observed with both human Pellino3 proteins but not the catalytically inactive mutants of hPellino3a and hPellino3b (Fig. 5b). In addition, the polyubiquitination of GSK3β protein was also found with wild-type Flag-mPellino3 but not the catalytically inactive mutant of mPellino3 (Fig. 5c). Unlike GSK3β, the MLK3 protein was not polyubiquitinated by hPellino3a and hPellino3b (Supplementary Fig. 4). These results indicated the possibility that both human and mouse Pellino3 specifically regulate APAP-induced liver injury through the polyubiquitination of GSK3β. Although we do not exclude the possibility that Pellino3 may modulate APAP hepatotoxicity through interaction with MLK3, we focused on the identification of the molecular mechanism of APAP-induced liver injury based on the Pellino3-mediated polyubiquitination of GSK3β. To further confirm the endogenous interaction of Pellino3 with GSK3β in mouse primary hepatocytes, we performed immunoprecipitation assays. However, we failed to confirm the endogenous interaction between the two proteins because all commercially available antibodies against Pellino3 did not detect endogenous Pellino3 expression. Despite these problems, we transfected the plasmid encoding Flag-mPellino3 into *Peli3*^−/−^ KO primary hepatocytes. After APAP treatment for the indicated durations, immunoprecipitation assays were performed with an anti-Flag antibody. Flag-Pellino3 protein ectopically expressed in *Peli3*^−/−^ KO hepatocytes specifically interacted with endogenous GSK3β at 2 h post APAP treatment (Fig. 5d).

Next, we examined whether the polyubiquitination of endogenous GSK3β is regulated by Pellino3 to verify the importance of Pellino3-mediated GSK3β polyubiquitination in APAP-induced liver injury. Hepatocytes were isolated from *Peli3*^+/+^ WT and *Peli3*^−/−^ KO mice, treated with APAP for the indicated times, and immunoprecipitated with anti-GSK3β antibody against endogenous GSK3β protein under 1% SDS denaturing conditions, and endogenous GSK3β polyubiquitination was observed by immunoblotting with anti-ubiquitin antibody. Increased polyubiquitination of endogenous GSK3β was observed in *Peli3*^+/+^ WT hepatocytes up to 4 h after APAP treatment (Fig. 5e). However, the polyubiquitination of endogenous GSK3β was significantly decreased in *Peli3*^−/−^ KO hepatocytes (Fig. 5e). Because the polyubiquitination process requires binding of an E3 ligase to a specific substrate, our results strongly indicate that Pellino3 is responsible for the polyubiquitination of GSK3β in APAP-induced liver injury through direct binding to GSK3β.

Based on these findings, we next investigated the polyubiquitination pattern of GSK3β by human Pellino3. Wild-type ubiquitin (HA-Ubi), the K48 ubiquitin mutant (HA-Ubi-K48) in which six lysine residues except lysine 48 are substituted into arginine, and the K63 ubiquitin mutant (HA-Ubi-K63) in which only lysine 63 is left intact were cotransfected into HEK293 cells with 6xMyc-GSK3β in the absence or presence of Flag-hPellino3a. K63- but not K48-linked GSK3β polyubiquitination was increased by Pellino3 (Fig. 5f). Because K63-linked polyubiquitination is known to be involved in various cellular activities, such as signal transduction, protein trafficking, protein‒protein interaction and DNA repair^51,52^, it is possible that K63-linked GSK3β polyubiquitination by Pellino3 influences GSK3β-mediated signal transduction in APAP-induced liver injury and does not affect the stability of the GSK3β protein. In fact, the expression of human Pellino3 did not dose-dependently affect GSK3β stability, and this process is mediated by K48-linked polyubiquitination (Fig. 5g).

### *Peli3* deficiency diminishes mitochondrial translocation and phosphorylation of GSK3β upon APAP treatment

Our present findings strongly indicate that Pellino3 acts as an E3 ligase to induce the polyubiquitination of GSK3β. GSK3β is constitutively active in the cytoplasm, and its activity is enhanced by phosphorylation at tyrosine 216 or inhibited by phosphorylation at serine 9 (Ser9)^53,54^. Interestingly, APAP treatment has been known to increase the phosphorylation of both active and inhibited forms of GSK3β and cause the translocation of both phosphorylated and total GSK3β proteins into mitochondria^32^. However, why the inhibited form of GSK3β is enhanced in APAP-mediated liver injury and how GSK3β is translocated into mitochondria remain unclear.

**Fig. 6:**
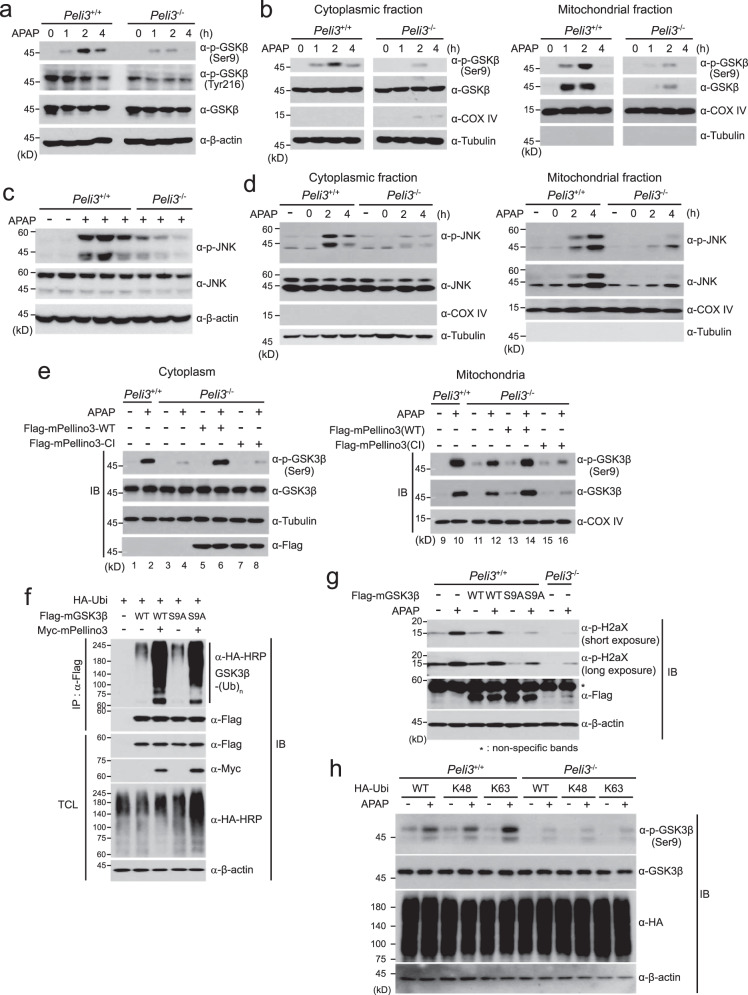
Pellino3 E3 ligase activity is needed for GSK3β phosphorylation and mitochondrial translocation. **a** Whole liver lysates isolated from *Peli3*^−/−^ and WT mice at the indicated times after the oral administration of 500 mg/kg APAP were immunoblotted with the indicated antibodies to examine the phosphorylation of GSK3β. **b** Primary hepatocytes isolated from *Peli3*^−/−^ and WT mice were treated with 20 mM APAP for the indicated times and subsequently fractionated into cytoplasmic and mitochondrial extracts. Both extracts were immunoblotted with the indicated antibodies against GSK3β phosphorylated at Ser9 and total GSK3β. The expression of tubulin and COX IV was used as cytoplasmic and mitochondrial markers and loading controls, respectively. **c** Whole liver lysates obtained from *Peli3*^−/−^ and WT mice at 4 h after the oral administration of 500 mg/kg APAP were immunoblotted with antibodies to detect the phosphorylation and total level of JNK. **d** Primary hepatocytes isolated from *Peli3*^−/−^ and WT mice were treated with 20 mM APAP for 2 h or 4 h and subsequently fractionated into cytoplasmic and mitochondrial extracts. Both extracts were immunoblotted with the indicated antibodies. **e** For rescue experiments, wild-type Flag-Pellino3 or a catalytically inactive (CI) mutant of Pellino3 was transfected into primary hepatocytes obtained from *Peli3*^−/−^ mice. As a control, a mock vector was transfected into primary hepatocytes obtained from wild-type mice. After both sets of hepatocytes were treated with 20 mM APAP for 2 h, they were fractionated into cytoplasmic and mitochondrial extracts and subsequently immunoblotted with the indicated antibodies to detect the phosphorylation of GSK3β at the Ser9 residue and the total levels of GSK3β. In (**d**) and (**e**), tubulin and COX IV were used as cytoplasmic and mitochondrial markers and loading controls. **f** Plasmids encoding wild-type Flag-mGSK3β, Flag-mGSKβ-S9A mutant and HA-Ubi were cotransfected into HEK293 cells together with a plasmid encoding Myc-mPellino3 according to the indicated combinations. GSK3β ubiquitination was examined by immunoprecipitation using an anti-Flag antibody and immunoblotting with an anti-HA-HRP antibody. Total cell lysates (TCLs) were immunoblotted with the indicated antibodies. **g** Primary hepatocytes of *Peli3*^+/+^ WT mice were transfected with wild-type Flag-mGSK3β or the Flag-mGSK3β-S9A mutant and subsequently treated with APAP for 8 h. Cell extracts were immunoblotted with anti-phospho-H2AX and anti-Flag antibodies. **h** After plasmids encoding HA-Ubi-WT, HA-Ubi-K48 and HA-Ubi-K63 were transfected into *Peli3*^+/+^ WT and *Peli3*^−/−^ KO hepatocytes according to the indicated combinations, the cells were treated with 20 mM APAP for 2 h, and cell extracts were immunoblotted with the indicated antibodies. **a**, **c**, **f**, **g**, **h** The expression of β-actin was used as a loading control. All immunoblot images in this figure are representative of at least three independent experiments.

Therefore, we next examined how Pellino3 modulates the function of GSK3β in APAP-induced liver injury. To this end, we investigated the phosphorylation and mitochondrial translocation of GSK3β in hepatocytes obtained from *Peli3*^+/+^ WT and *Peli3*^−/−^ KO mice that were orally administered APAP. The level of phosphorylation at Ser9 of GSK3β was significantly decreased in *Peli3*^−/−^ KO mice, whereas the phosphorylation of tyrosine 216 (Tyr216) was hardly affected by *Peli3* deficiency (Fig. 6a). Cytoplasmic and mitochondrial fractionation assays revealed that the mitochondrial translocation of both total GSK3β and GSK3β phosphorylated at Ser9 was initiated at 1 h post APAP treatment in *Peli3*^+/+^ WT hepatocytes. In contrast, the mitochondrial translocation of GSK3β and phosphorylation at Ser9 were significantly decreased in *Peli3*^−/−^ KO mice compared to *Peli3*^+/+^ WT mice (Fig. 6b). However, the reduced phosphorylation of GSK3β at Ser9 in the mitochondrial fraction of *Peli3*^−/−^ hepatocytes seems to be due to decreased mitochondrial translocation of total GSK3β.

Next, we investigated the effect of *Peli3* deficiency on the activation of JNK in APAP-induced liver injury because it has been reported that JNK activation is downstream of GSK3β during APAP hepatotoxicity and JNK is also translocated into mitochondria^27,34^. After hepatocytes were isolated from independent *Peli3*^+/+^ WT (*n* = 5) and *Peli3*^−/−^ KO (*n* = 3) mice that were orally administered APAP, we analyzed the phosphorylation of JNK by immunoblotting. Upon APAP treatment, the phosphorylation of JNK was increased in three independent *Peli3*^+/+^ WT mice and profoundly decreased in *Peli3*^−/−^ KO mice (Fig. 6c). In addition, the mitochondrial translocation of phosphorylated JNK and total JNK from the cytoplasm was significantly decreased upon APAP treatment in *Peli3*^*−/−*^ KO mice compared to *Peli3*^+/+^ WT mice (Fig. 6d). Similar to the results found for GSK3β, the reduced phosphorylation of JNK in the mitochondrial fraction of *Peli3*^−/−^ hepatocytes seems to be caused by decreased mitochondrial translocation of JNK. These results collectively imply that Pellino3 is an upstream signaling component regulating the phosphorylation and mitochondrial translocation of GSK3β, which subsequently regulates JNK phosphorylation in the cytoplasm and the mitochondrial translocation of JNK in APAP-induced liver injury.

We next performed rescue experiments to clearly assess the importance of the E3 ligase activity of Pellino3 in the phosphorylation and mitochondrial translocation of GSK3β. Hepatocytes obtained from *Peli3*^−/−^ KO mice as well as and *Peli3*^+/+^ WT hepatocytes were transfected with wild-type mFlag-Pellino3 or a catalytically inactive mutant of Pellino3 (Flag-mPellino3-CI) respectively, the hepatocytes were treated with APAP for 2 h and subsequently separated into cytoplasmic and mitochondrial fractions. Similar to our previous results (Fig. 6a, b), GSK3β mitochondrial translocation and Ser9 phosphorylation were decreased in *Peli3*^−/−^ KO hepatocytes compared to *Peli3*^+/+^ WT hepatocytes (Fig. 6e; Lanes 2, 4, 10, 12). Interestingly, the ectopic expression of wild-type mPellino3 in *Peli3*^−/−^ KO hepatocytes significantly restored the mitochondrial translocation and Ser9 phosphorylation of GSK3β (Fig. 6e; Lanes 4, 6, 12, 14), whereas the expression of the catalytically inactive mutant of Pellino3 (Flag-mPellino3-CI) did not yield this effect (Fig. 6e; Lanes 6, 8, 14). These results suggest that the E3 ligase activity of Pellino3 is crucial for the regulation of GSK3β in APAP-induced liver injury.

Although our present findings indicate that GSK3β Ser9 phosphorylation requires the E3 ligase activity of Pellino3 (Fig. 6a, b), it is possible that the phosphorylation of GSK3β at Ser9 may be necessary for the Pellion3-mediated ubiquitination of GSK3β. To test this possibility, we examined the ubiquitination of the GSK3β-S9A mutant, in which the Ser9 residue was substituted with alanine. The GSK3β-S9A mutant was polyubiquitinated by wild-type Pellino3 protein, similar to wild-type GSK3β (Fig. 6f). Taken together with our current findings, these results indicate that the Pellino3-mediated ubiquitination of GSK3β is upstream of GSK3β Ser9 phosphorylation, which is essential for APAP hepatotoxicity, and the phosphorylation of GSK3β at Ser9 is not a prerequisite for GSK3β ubiquitination by Pellino3. In fact, the importance of the phosphorylation of GSK3β at Ser9 in APAP hepatotoxicity was confirmed by the results that ectopic expression of the GSK3β-S9A mutant in *Peli3*^+/+^ WT hepatocytes did not increase the phosphorylation of histone H2AX upon APAP treatment (Fig. 6g).

Furthermore, we investigated the importance of the K63-linked polyubiquitination of GSK3β by Pellino3 in GSK3β phosphorylation at Ser9 and its mitochondrial translocation. Plasmids encoding the K48 (HA-Ubi-K48) or K63 (HA-Ubi-K63) ubiquitin mutant were transiently transfected into *Peli3*^+/+^ WT or *Peli3*^−/−^ KO primary hepatocytes, and the cells were subsequently treated with APAP for 2 h. As a control, wild-type ubiquitin (HA-Ubi-WT) was also transfected into primary hepatocytes. The phosphorylation of GSK3β at Ser9 and its mitochondrial translocation in *Peli3*^+/+^ WT hepatocytes were basally increased by APAP treatment in the presence of wild-type ubiquitin (HA-Ubi-WT) and the K48 ubiquitin mutant (HA-Ubi-K48) (Fig. 6h and Supplementary Fig. 5). In contrast, expression of the K63 ubiquitin mutant (HA-Ubi-K63) further increased the phosphorylation of endogenous GSK3β at Ser9 and its mitochondrial translocation by APAP treatment compared with the results obtained with HA-Ubi-WT and HA-Ubi-K48 (Fig. 6h and Supplementary Fig. 5). This significant increase in GSK3β phosphorylation at Ser9 and its mitochondrial translocation in the presence of HA-Ubi-K63 was not observed in *Peli3*^−/−^ KO primary hepatocytes (Fig. 6h and Supplementary Fig. 5). Therefore, these results support our current finding that the K63-linked polyubiquitination of GSK3β by Pellino3 is needed for GSK3β phosphorylation at Ser9 and mitochondrial translocation in APAP-induced liver injury.

## Discussion

Although the pathophysiology of APAP-mediated liver injury and the signaling pathways governing this hepatotoxicity have been extensively studied over the past decades, we do not have a clear understanding of the ubiquitin-modifying systems that regulate posttranslational modification of signaling components in APAP-induced liver injury. Only a few studies have suggested that E3 ligases such as endoplasmic reticulum-polytopic gp78/AMFR (autocrine motility factor receptor), Parkin and HOIP are involved in APAP-induced liver injury by using knockout mice or RNA interference technology^55–57^.

In this study, we demonstrated that the Pellino3 protein is a novel E3 ubiquitin ligase in APAP-induced liver injury and that the K63-linked polyubiquitination of GSK3β by Pellino3 leads to GSK3β phosphorylation at Ser9 in the cytoplasm and mitochondrial translocation. Phosphorylated GSK3β contributes to augmentation of the levels of mitochondrial ROS, mitochondrial dysfunction and DNA damage, which eventually results in necrotic cell death (Supplementary Fig. 6). In addition, the findings that *Peli3* mRNA expression is hardly affected by APAP treatment indicate that the interaction of Pellino3 with GSK3β is more important than the regulation of *Peli3* mRNA in APAP hepatotoxicity. Thus, this is the first study to identify Pellino3 as an E3 ubiquitin ligase targeting GSK3β in hepatocytes and reveal its pathophysiological role in *Peli3*^*−/−*^ KO mice orally administered APAP.

In this study, we hypothesized that Pellino3 may play an important role in APAP-induced liver injury because Pellino3 has been identified to promote the activation of c-Jun and Elk-1 and is involved in diverse inflammatory signaling pathways by targeting different substrates^25,43^. Herein, we clearly showed reduced APAP-induced liver injury in both whole-body *Peli3*^−/−^ KO and hepatocyte-specific *Peli3*-depleted mice. This protective effect of *Peli3* deficiency in APAP hepatotoxicity seems to be due to decreases in the phosphorylation and mitochondrial translocation of GSK3β accompanied by reductions in mitochondrial ROS and dysfunction and DNA damage. In fact, mounting evidence indicates that the disastrous changes caused by APAP treatment require GSK3β and JNK phosphorylation and their mitochondrial translocation, and GSK3β is upstream of JNK^32,58^. Our current findings with *Peli3*^−/−^ KO mice provide robust evidence showing that the Pellino3-mediated ubiquitination of GSK3β in hepatocytes is an essential step for the phosphorylation of GSK3β at Ser9 and GSK3β mitochondrial translocation, which are critical for mitochondrial damage in APAP hepatoxicity. However, we could not exclude the possibility that Pellino3 in inflammatory cells contributes to APAP hepatotoxicity in an unidentified manner because Pellino3 has been reported to be involved in diverse inflammatory signaling pathways.

Although GSK3β has received attention as an important regulator of mitochondrial activity and APAP treatment in mice is known to cause GSK3β activation and mitochondrial translocation^32,58^, we still do not know the exact mechanism of mitochondrial translocation of GSK3β in hepatocytes. A clue to how GSK3β is translocated into mitochondria may be derived from previously reported experiments in H9c2 cardiomyoblasts^59^. In these cells, GSK3β translocates to mitochondria under oxidative stress by interacting with voltage-dependent anion channel 2 (VDAC2) in a manner dependent on the phosphorylation of GSK3β at Ser9^59^. Based on this previous finding, the K63-linked polyubiquitination of GSK3β by Pellino3 may be crucial for the interaction of GSK3β with a certain factor on the mitochondrial membrane in APAP-induced liver injury. Therefore, our study may provide clues for further expansion of our knowledge of the mechanism of the mitochondrial translocation of GSK3β upon APAP treatment.

However, the functions of Pellino3 in GSK3β are likely to expand beyond the mitochondrial translocation of GSK3β. Compared to *Peli3*^+/+^ WT mice, GSK3β Ser9 phosphorylation in the cytoplasm was significantly decreased in *Peli3*^−/−^ KO mice, whereas a change in tyrosine 216 phosphorylation was not detected. In general, GSK3β activity is known to be enhanced by tyrosine 216 phosphorylation or inhibited by Ser9 phosphorylation. However, APAP treatment reportedly increases the phosphorylation of both the active and inhibited forms of GSK3β, even though the reason has not been addressed until now^32^. Because kinases that directly phosphorylate GSK3β at Ser9 have not been reported, it is possible that the E3 ligase activity of Pellino3 regulates certain kinases that control GSK3β phosphorylation at Ser9. In this respect, it is notable that the expression of GSK3β and the phosphorylation of glycogen synthase (GS), which is the substrate of GSK3beta, were decreased in the liver extracts of MLK3 KO mice upon APAP treatment, which implies that MLK3 is connected to GSK3β through a positive feedback loop in APAP-induced liver injury^33^. However, there is no direct evidence showing that MLK3 is a kinase that phosphorylates GSK3β at Ser9. Considering our finding that Pellino3 binds to MLK3 independent of its E3 ligase activity, it is worth investigating whether the K63-linked polyubiquitination of GSK3β by Pellino3 promotes GSK3β phosphorylation at Ser9 by MLK3 or an unknown kinase. Based on a summary of findings and those detailed in previous reports, the Pellino3-mediated K63-linked polyubiquitination of GSK3β is critically related to Ser9 phosphorylation of GSK3β in the cytoplasm, and its phosphorylation at Ser9 promotes the mitochondrial translocation of GSK3β in APAP hepatotoxicity. This speculation may be partly supported by a previous finding that the mitochondrial translocation of GSK3β in H9c2 cardiomyoblasts is dependent on Ser9 phosphorylation^59^.

Additionally, our results that *Peli3* deficiency reduces ROS generation and hepatic GSH levels strongly indicate that Pellino3 may be involved in APAP-induced oxidative stress in an unidentified manner. Thus, it is possible that Pellino3 may be related to the expression of antioxidant enzymes such as superoxide dismutase 2 (SOD2). Therefore, the underlying mechanism through which Pellino3 specifically regulates the expression of antioxidant enzymes such SOD2 in APAP hepatotoxicity should be a topic of future interest.

Our findings that Pellino3 binds to the MAP kinases GSK3β, MLK3, and JNK1 with different binding strengths imply that Pellino3 may have scaffolding activity in the signaling pathway regulating APAP hepatotoxicity. This speculation would be consistent with the previous finding that Pellino3 acts as a scaffolding protein^43^. In addition, our study showed that the increased survival rate of mice orally administered APAP correlates with the reduction in endogenous *Peli3* mRNA expression by recombinant adenoviruses expressing *Peli3*-specific shRNAs, which suggests that *Peli3* depletion after the onset of APAP hepatotoxicity is able to reduce liver damage. This finding emphasizes the importance of the Pellino3-GSK3β axis on the initiation and progression of APAP hepatoxicity. In fact, our present findings show that the Pellino3-mediated ubiquitination of GSK3β, which leads to the phosphorylation of GSK3β at Ser9 and mitochondrial translocation of GSK3β, is critical for the pathological process of APAP-induced liver injury, and that *Peli3* deficiency decreases mitochondrial ROS and damage as well as lysosomal damage. Conclusively, these findings strongly suggest that the Pellino3-GSK3β axis in hepatocytes is crucial for APAP-induced liver injury, emphasizing that the blockade of GSK3β ubiquitination by Pellino3 could be an important regimen for the treatment of APAP hepatotoxicity.

## Supplementary information

Supplementary information
