# Supplementary material for: Peli3 ablation ameliorates acetaminophen-induced liver injury through inhibition of GSK3β phosphorylation and mitochondrial translocation
Source: Exp Mol Med. 2023 Jun 1;55(6):1218–31. doi: 10.1038/s12276-023-01009-w (PMC10318043; doi:10.1038/s12276-023-01009-w)
Supplement: Supplementary file 1 — Supplementary information [file 12276_2023_1009_MOESM1_ESM.pdf]

Supplementary Information

Peli3 ablation ameliorates acetaminophen-induced liver injury through inhibition of GSK3β phosphorylation and mitochondrial translocation

Jaewon Lee, Jihoon Ha, Jun-Hyeong Kim, Dongyeob Seo, Minbeom Kim, Yerin Lee, Seong Shil Park, Dahee Choi, Jin Seok Park, Young Jae Lee, Siyoung Yang, Kyung-Min Yang, Su Myung Jung, Suntaek Hong, Seung-Hoi Koo, Yong-Soo Bae, Seong-Jin Kim, Seok Hee Park

<Table of Contents>

1. Supplementary materials and methods
2. Supplementary figure 1 – 6
3. Supplementary Table 1 – 4

Supplementary materials and methods

Histological analysis, neutrophil infiltration and TUNEL assay

Liver tissue was fixed in 10 % neutral buffered formalin (NBF) solution for 24 h and embedded in paraffin. Hematoxylin and eosin (H&E) staining for morphological analysis was performed on 5 μm thick tissue sections. To detect the infiltration of neutrophils, NIMP antibody against Ly6G/Ly6C was used for immunohistochemistry and myeloperoxidase (MPO) activities were measured with 20 mg lysates of livers by the MOUSE MPO ELISA kit (Thermo Fisher Scientific), according to the manufacturer's instructions. For terminal deoxynucleotidyl transferase-mediated dUTP nick-end labeling (TUNEL) assay, frozen sections of liver tissues were stained by the *In Situ* Cell Death Detection Kit, POD (Roche, Mannheim, Germany, catalog #; 11 684 817 910), according to the manufacturer's instructions.

Primary hepatocyte isolation

Hepatocytes were isolated by liver perfusion from 8- to 10-week-old male mice. Mice were anesthetized with isoflurane, and 18 ml of Perfusion solution I (0.142 M NaCl, 6.7m M KCl, 10 mM HEPES, 0.247 M EGTA, pH 7.4) and 45 ml of Perfusion solution II (66.7 mM NaCl, 6.7 mM KCl, 50 mM HEPES, 4.8 mM $CaCl_2 \cdot 2H_2O$, 5 g/L BSA, 0.4 g/L collagenase IV, pH 7.6) were sequentially used for liver perfusion. Solutions were perfused through the portal vein for 2 min (solution I), and for 5 min (solution II), at a flow rate of 9 ml/min. After collagenase digestion was finished, the liver was minced and filtrated through a 70 μm cell strainer. Primary hepatocytes were separated by centrifugation at 500 rpm for 4 min at 4 °C and subsequently purified with 40 % Percoll through centrifugation at 1,250 rpm for 5min.

Glutathione (GSH) and reactive oxygen species (ROS) measurements

Oxidized GSH (glutathione disulfide, GSSG) and total GSH were measured by the Glutathione (GSSG/GSH) Detection kit (Enzo Life Science, Farmingdale, NY), according to the manufacturer's instructions. Briefly, liver tissues were homogenized in ice-cold 5 % (w/v) metaphosphoric acid (Sigma-Aldrich), followed by centrifugation at 12,000 rpm for 10 min at 4 °C to remove proteins which interfere with the assay. The resultant supernatant was collected for glutathione detection and transferred to 96-well plates. 2 M 4-vinylpyridine (Sigma-Aldrich) was used for blocking free thiols present in the reaction, eliminating any contribution to the cycling reaction caused by GSH. Absorbance at 405 nm was observed by a microplate reader at 2 min intervals for 20 min. Total intracellular ROS in primary hepatocytes was stained by 2',7'–dichlorofluorescein diacetate (H_2DCFDA) and measured at 488 nm using FACSCanto II (BD Bioscience, Franklin Lakes, NJ). Mitochondrial ROS was measured using MitoSOX™ Red (Thermo Fisher Scientific, M36008), according to the manufacturer's instructions. After APAP treatment for 4 h, primary hepatocytes were trypsinized and collected by centrifugation. The cells were resuspended in a solution containing 5 µM MitoSOX reagent, incubated for 10 min, washed with PBS and analyzed by Northern Lights (Cytek Biosciences, Fremont, CA). The data regarding intracellular and mitochondrial ROS were analyzed by FlowJo 7.6.5 software.

MTT and lysosomal activity assays

To evaluate mitochondrial and lysosomal function, MTT and lysosomal activity assays were performed. Primary hepatocytes obtained from *Peli3*$^{-/-}$ KO and *Peli3*$^{+/+}$ WT mice were cultured in six-well plates with 4 x 10^5 cells. MTT solution (Roche, 11465007001) was added to each well and incubated for 20 m at 37 °C according to the manufacturer's instructions. The media was discarded and 2 mL of DMSO was subsequently added. Absorbance values at 590 nm were determined by a Bio-Rad 680 microplate reader (Bio-Rad). For lysosomal activity assays,

primary hepatocytes were treated with APAP for 8 h, lysosomal activity was measured with a self-quenched substrate (FITC), together with bafilomycin 1x as a control, following the manufacturer's instructions of the Lysosomal Intracellular Activity Assay kit (Abcam, Cambridge, UK; ab234622). The signal was quantified by Northern Lights (Cytek). The data were analyzed by FlowJo 7.6.5 software.

Fractionation of cytosolic and mitochondrial extracts

Cytosolic and mitochondrial extracts were fractionated by differential centrifugation using the Mitochondria Isolation kit (Thermo Fisher Scientific), according to the manufacturer's instructions. Protease inhibitor cocktail, sodium fluoride (NaF), and sodium orthovanadate (NaOV) were further added for detection of phosphorylated proteins. The degree of cytosolic and mitochondrial fractionation was assessed by expressions of tubulin and cytochrome c oxidase subunit 4 (COX4), respectively. Both cytosolic and mitochondrial extracts were stored at -80 °C until analysis.

Immunoblotting, immunoprecipitation and *in vivo* ubiquitination assay

Immunoblotting and immunoprecipitation assays were performed as described previously[49]. *In vivo* ubiquitination assay was performed as described previously[47]. For the ubiquitination assay, non-covalent interactions of proteins were dissociated by 1 % SDS and boiling for 10 min. Samples were diluted ten times with lysis buffer (PBS containing 0.5 % Triton X-100, 20 mM HEPES (pH 7.4), 150 mM NaCl, 12.5 mM β-glycerol phosphate, 1.5 mM MgCl2, 10 mM NaF, 2 mM DTT, 1 mM NaOV, 2 mM EGTA, 1 mM PMSF, and protease inhibitor cocktail) and subsequently suspended using a 1 ml syringe. Samples were pre-cleared by protein agarose G beads and immunoprecipitated by the indicated antibodies.

ELISA and MPO assays

The levels of the pro-inflammatory cytokines IL-1β, IL-6, TNFα in the blood serum were measured by an ELISA kit (eBioscience, Waltham, MA). Myeloperoxidase (MPO) level was detected using the commercial MOUSE MPO ELISA KIT (Hycult Biotech, Uden, Netherlands). Twenty mg of liver tissues were homogenized and lysed in 400 μl lysis buffer [200 mM NaCl, 5 mM EDTA, 10 mM Tris, 10 % glycerin, 1 mM PMSF, 1 μg/ml leupeptin, 28 μg/ml aprotinin (pH 7.4)]. Samples were centrifuged twice to avoid contamination of cell debris. The levels of all cytokines and myeloperoxidase were measured at 450 nm wavelength.

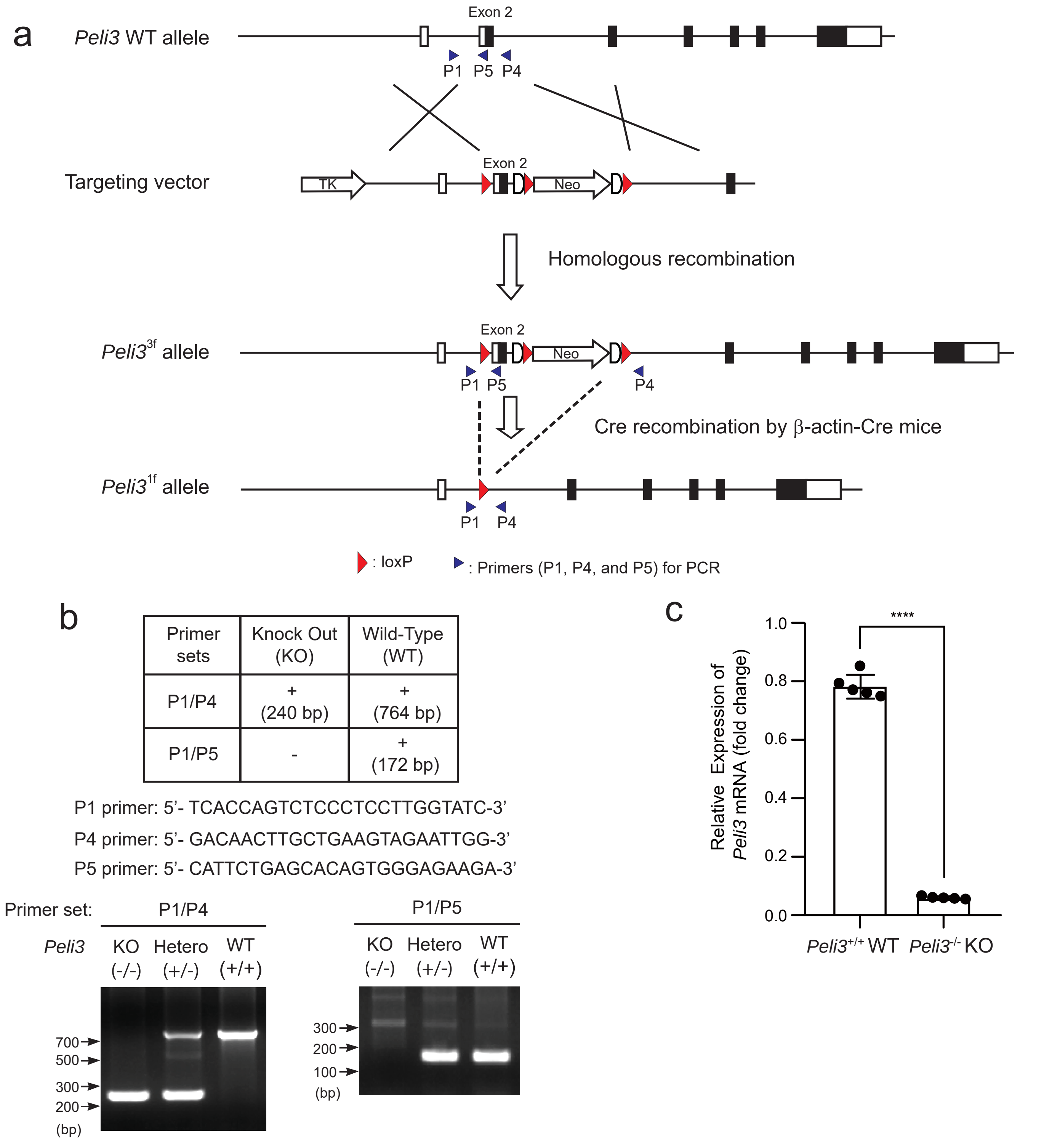

Primer sets	Knock Out (KO)	Wild-Type (WT)
P1/P4	+ (240 bp)	+ (764 bp)
P1/P5	-	+ (172 bp)

P1 primer: 5'- TCACCAGTCTCCCTCCTTGGTATC-3'

P4 primer: 5'- GACAACTTGCTGAAGTAGAATTGG-3'

P5 primer: 5'- CATTCTGAGCACAGTGGGAGAAGA-3'

Supplementary Fig. 1. Generation of the *Peli3* knockout allele
(**a**) Schematic diagram of the *Peli3* wild-type (WT), targeted (*Peli3*^{3f}), and null (*Peli3*^{1f}) alleles. Exons are represented by boxes and the coding region is indicated by black boxes. Frt sequences and loxP sequences flanking a neo cassette (Neo) or exon2 (E2) are indicated by semicircles and red triangles, respectively. Locations of primers (P1, P4, P5) used for genotyping are indicated by blue arrowheads. (**b**) Genotyping results of *Peli3*$^{-/-}$ KO and *Peli3*$^{+/+}$ WT mice by polymerase chain reaction (PCR). The genotyping results are representative of at least three independent experiments. (**c**) Relative expression of *Peli3* mRNA isolated from the tails of *Peli3*$^{-/-}$ KO and *Peli3*$^{+/+}$ WT mice by quantitative real-time reverse transcriptase-PCR (qRT-PCR). n=3 per group. The data were statistically analyzed by two-way ANOVA followed by Sidak's multiple comparison test (****$P < 0.0001$ compared to the control groups, *Peli3*$^{+/+}$ WT mice). The bars represent the mean ± SD.

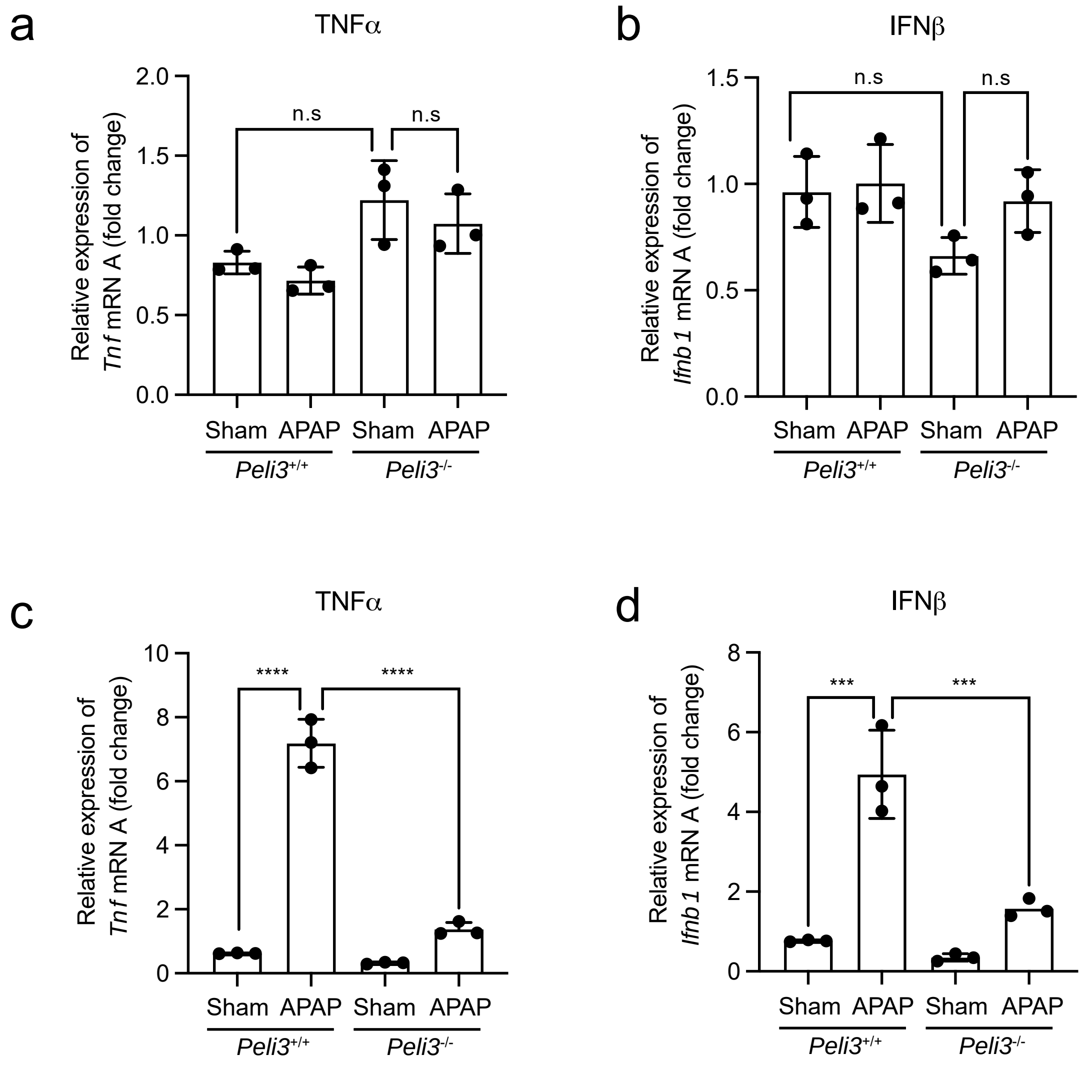

Supplementary Fig. 2. Expressions of TNFα and IFNβ are decreased in whole liver extracts of *Peli3$^{-/-}$* KO mice.

Expressions of *Tnf* and *IFNb1* mRNAs in primary hepatocytes (**a** and **b**) and whole liver extracts (**c** and **d**) of *Peli3$^{-/-}$* KO and *Peli3$^{+/+}$* WT mice were examined by real-time qRT-PCR. n=3 per group. The data were statistically analyzed by two-way ANOVA followed by Sidak's multiple comparison test (***P < 0.001, ****P < 0.0001 compared to the control groups, *Peli3$^{+/+}$* WT mice). n.s; not significant. The bars represent the mean ± SD.

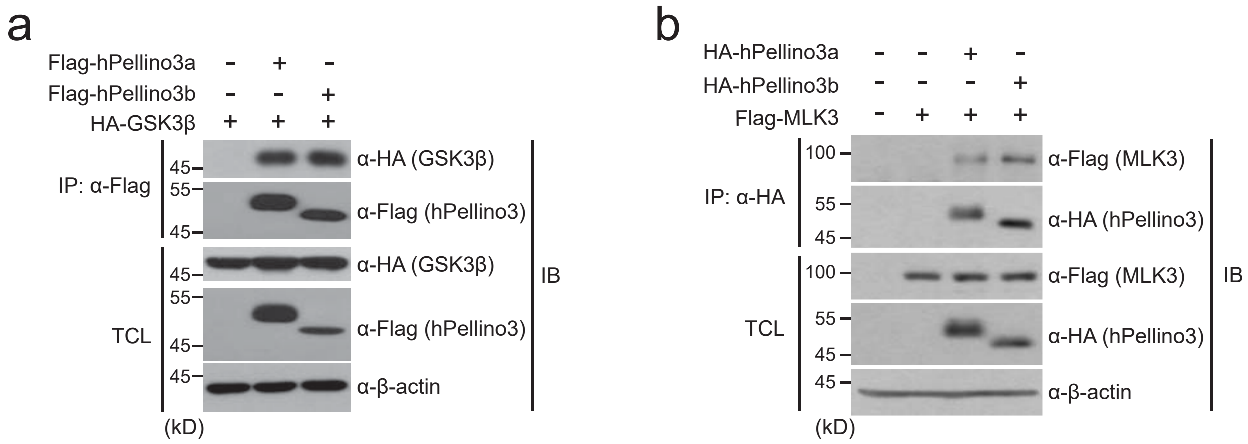

Supplementary Fig. 3. Both Pellino3a and Pellino3b bind to GSK3β and MLK3, respectively. (**a**) A plasmid encoding HA-GSK3β was co-transfected into HEK293 cells with Flag-Pellino3a or Flag-Pellino3b. Cell lysates were immunoprecipitated (IP) with anti-Flag antibody and subsequently immunoblotted (IB) with anti-HA and anti-Flag antibodies. (**b**) A plasmid encoding Flag-MLK3 was co-transfected into HEK293 cells with HA-Pellino3a or HA-Pellino3b. Cell lysates were immunoprecipitated (IP) with anti-HA antibody and subsequently immunoblotted (IB) with anti-Flag and anti-HA antibodies. In (**a**) and (**b**), total cell lysates (TCL) were immunoblotted with the indicated antibodies and expression of β-actin was used as a loading control. Immunoblot images in this figure are representative of at least three independent experiments.

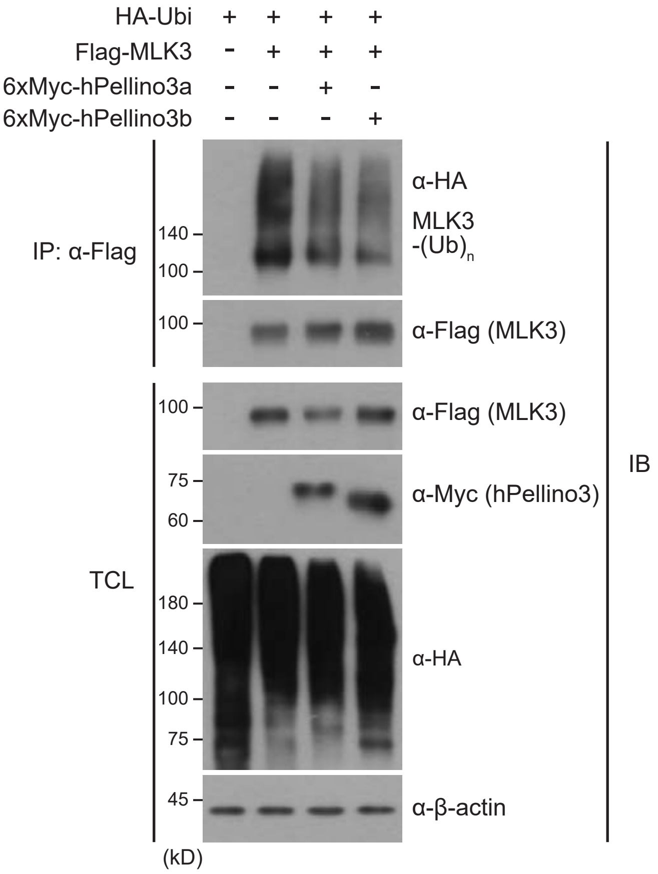

Supplementary Fig. 4. Pellino3 does not induce polyubiquitination of the MLK3 protein. Plasmids encoding Flag-MLK3 and HA-Ubi were co-transfected into HEK293 cells together with a plasmid encoding wild-type 6xMyc-Pellino3a or 6xMyc-Pellino3b. MLK3 ubiquitination was examined by immunoprecipitation using anti-Myc antibody and immunoblotting with anti-HA and anti-Flag antibodies. Total cell lysates (TCL) were immunoblotted with the indicated antibodies. Expression of β-actin was used as a loading control. The immunoblot image is representative of at least three independent experiments.

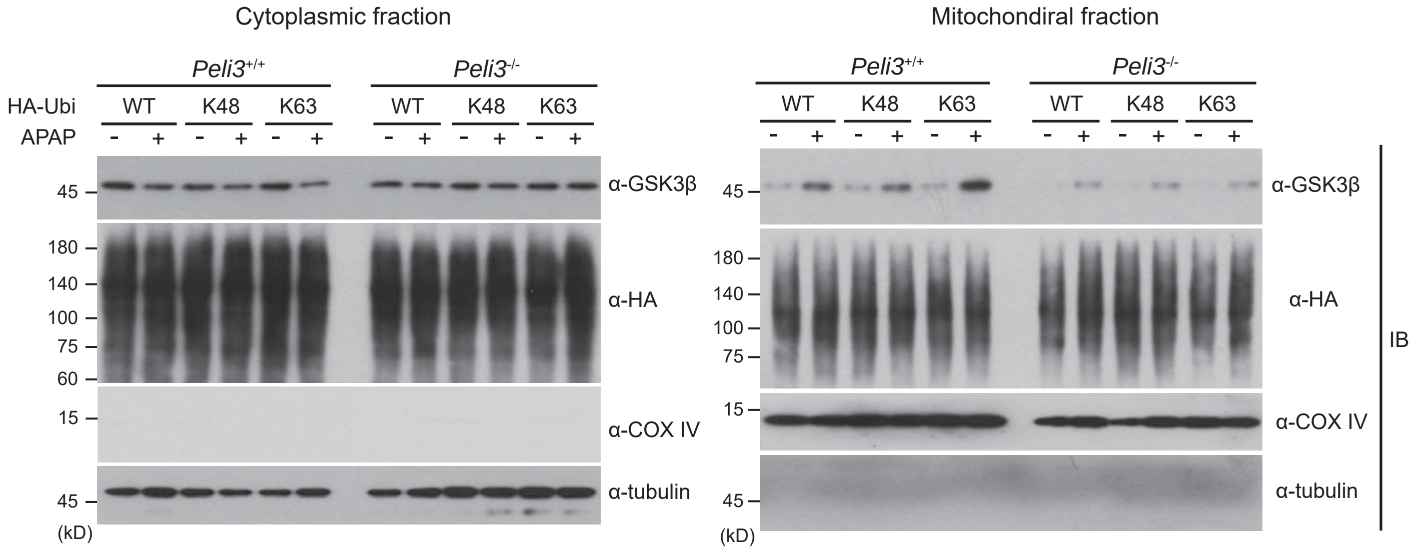

Supplementary Fig. 5. Ectopic expression of the K63 ubiquitin mutant increases mitochondrial translocation of GSK3β in *Peli3*$^{+/+}$ WT hepatocytes, but not *Peli3*$^{-/-}$ KO.

After plasmids encoding HA-Ubi-WT, HA-Ubi-K48, and HA-Ubi-K63 were transfected into *Peli3*$^{+/+}$ WT and *Peli3*$^{-/-}$ KO hepatocytes, accorording to the indicated comibinations, and subsequently treated with 20 mM APAP for 2 h. Cells were fractionated into cytoplasmic and mitochondrial extracts. Both extracts were immunoblotted with the indicated antibodies. Expression of tublin and COX IV were used respectively as cytoplasmic and mitochondrial markeres and loading controls. The immunoblot image is representative of at least three independent experiments.

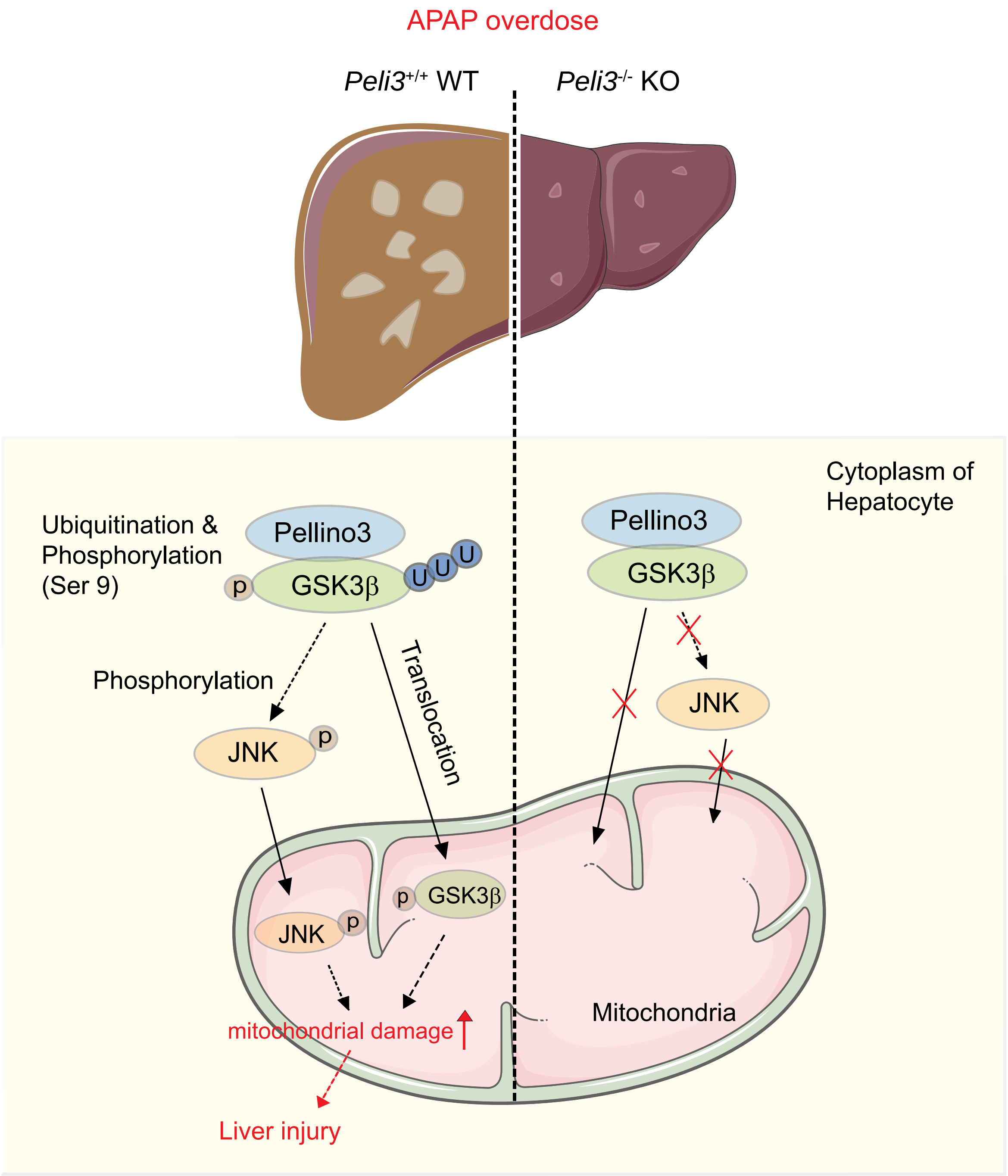

Supplementary Fig. 6. The proposed mechanism of APAP-induced liver injury regulated by the Pellino3-GSK3β axis.
Upon APAP treatment, Pellino3 protein with RING type E3 ubiquitin ligase activity binds to GSK3β and induces K63-linked polyubiquitination of GSK3β. This polyubiquitination subsequently leads to phosphorylation at serine 9 in the cytoplasm and facilitates mitochondrial translocation of GSK3β. In addition, GSK3β activated by Pellino3-mediated polyubiquitination increases the phosphorylation and mitochondrial translocation of JNKs, which are downstream kinases of GSK3β. This GSK3β and JNK activation by Pellino3 contributes to the augmentation of mitochondrial ROS and mitochondrial and lysosomal damage, eventually resulting in APAP-induced liver injury.

Supplementary Table 1. Primer sequences for real-time quantitative RT-PCRs used in this study.

Construct	Species	Direction	Sequence (5` → 3`)
Peli3	Mouse	Forward	CGATGAGCGAACCGTCTTG
		Reverse	CTGTCTGCGGGAATGTGTA
Peli3 (For genotyping)	Mouse	Forward	TCACCAGTCTCCCTCCTTGGTATC
		Reverse 1	GACAACTTGCTGAAGTAGAATTGG
		Reverse 2	CATTCTGAGCACAGTGGGAGAAGA
Tnf	Mouse	Forward	GGACTAGCCAGGAGGGAGAA
		Reverse	CGCGGATCATGCTTTCTGTG
Ifnb1	Mouse	Forward	CAGCCTGGCTTCTACATGATGA
		Reverse	TTCCATTCAGCTGCTCCAG

Supplementary Table 2. The target sequences of RNAi used to construct recombinant adenoviruses in this study.

**shRNA**	**Species**	**Target Sequence**
sh*Peli3 #3*	Mouse	CUGAGAAGACUGCCCGCUA
si*Peli3 #4*	Mouse	AACUAGAGGCCCAGCGACA

Supplementary Table 3. Primer sequences used to construct plasmids in this study.

Construct	Direction	Sequence (5' → 3')
Flag-GSK3β	Forward	CCGGAATTCATGTCAGGGCGGCCCAGA
	Reverse	CATGCCATGGCGGTGGAGTTGGAAGCTGATG
6xMyc-GSK3β	Forward	CCGGAATTCATGTCAGGGCGGCCCAGA
	Reverse	ACGCGTCGACTCAGGTGGAGTTGGAAGCT
Flag-JNK1	Forward	CCGGATATCATGAGCAGA AGCAAACGTGAC
	Reverse	CCGCTCGAGTCATCTACAG CAGCCCAGAGG
Flag-JNK2	Forward	CCGGAATTCATGAGTGA CAGTAAAAGCGATG
	Reverse	CCGCTCGAGTCACCGGCAG CCTTCCAG
Flag-MKK4	Forward	CGGGATCCATGGCGGCTCCGAGCCC
	Reverse	CCGCTCGAGTCAATCGACATACATGGGAGA
Flag-MKK7	Forward	CGGAATTCATGGCGGCGTCCTCCCTG
	Reverse	CCGCTCGAGCTACCTGAAGAAGGGCAGGT
Flag-MLK3	Forward	CGGAATTCATGGAGCCCTTGAAGAGCCT
	Reverse	CCGCTCGAGTCAAGGCCCCGCTTCCGG
Flag-hPellino3a	Forward	CCGGAATTCATGGTGCTGGAAGGAAACC
	Reverse	CCGCTCGAGCTAATCCAGCGGGCCCTGGAAA
Flag-hPellino3b	Forward	CCGGAATTCATGGTGCTGGAAGGAAACC
	Reverse	CCGCTCGAGCTAATCCAGCGGGCCCTGGAA
Flag-hPellino3a-CI (C384A, C387A)	Forward	CCCCCAGGAGCGCGAAGCTCCTCTCGCCCGCCTTGTG
	Reverse	CACAAGGCGGGCGAGAGGAGCTTCGCGCTCCTGGGGG
Flag-hPellino3b-CI (C360A, C363A)	Forward	CCCCCAGGAGCGCGAAGCTCCTCTCGCCCGCCTTGTG
	Reverse	CACAAGGCGGGCGAGAGGAGCTTCGCGCTCCTGGGGG
6xMyc-hPellino3a	Forward	CCGCTCGAGAATGGTGCTGGAAGGAAACC
	Reverse	CGGGGTACCCTAATCCAGCGGGCCCTGGA
6xMyc-hPellino3b	Forward	CCGCTCGAGAATGGTGCTGGAAGGAAACC
	Reverse	CGGGGTACCCTAATCCAGCGGGCCCTGGA
Flag-mPellino3	Forward	CCGGAATTCATGGTGCTGGAAGGAAACC

	Reverse	CCGCTCGAGCTAGTCCAGTGGCCCCTG
Flag-mPelino3-CI (C360A, C363A)	Forward	CCCCAGGAGCGAGAGGCTCCTCTCGCCCGCCTTGTGGGACCC
	Reverse	GGGTCCCACAAGGCGGGCGAGAGGAGCCTCTCGCTCCTGGGG
Flag-mGSK3β	Forward	CCGGATATCGATGTCGGGGCGACCGAGA
	Reverse	CATGCCATGGCGGTGGAGTTGGAAGCTGATG
Flag-mGSK3β (S9A)	Forward	CGACCGAGAACCACCGCCTTTGCGGAGAGCTGC
	Reverse	GCAGCTCTCCGCAAAGGCGGTGGTTCTCGGTCG
6xMyc-mPellino3	Forward	CCGCTCGAGAATGGTGCTGGAAGGAAACC
	Reverse	ACGCGTCGACCTAGTCCAGTGGCCCCTG

Supplementary Table 4. Antibodies used in this study.

Antibody Name	Company Name	Catalog Number	Species	Assay	Dilution/Amount	Validation
JNK	Cell Signaling Technology	9252	Rabbit	IB	1:2000	Datasheet by company
p-JNK	Cell Signaling Technology	9251	Rabbit	IB	1:1000	Datasheet by company
COX IV	Cell Signaling Technology	4850	Rabbit	IB	1:10000	Datasheet by company
SOD2	Cell Signaling Technology	13141	Rabbit	IB	1:10000	Datasheet by company
GSK3β	Cell Signaling Technology	12456	Rabbit	IB, IP	1:1000 for IB, 3μg for IP	Datasheet by company
p-H2AX	Cell Signaling Technology	9718	Rabbit	IB	1:2000	Datasheet by company
GSK3β	Santa Cruz	sc-9166	Rabbit	IB	1:2000	Datasheet by company
p-GSK3β (Tyr216)	BD	612312	Mouse	IB	1:1000	Datasheet by company
p-GSK3β (Tyr216)	Santa Cruz	sc-11758	Goat	IB	1:1000	Datasheet by company
p-GSK3β (Ser9)	Cell Signaling Technology	5558	Rabbit	IB	1:1000	Datasheet by company
NIMP	Abcam	ab2577	Mouse	IHC	1:100	Datasheet by company
c-Myc	Santa Cruz	sc-40	Mouse	IB, IP	1:5000 for IB, 1μg for IP	Datasheet by company
Myc-HRP	Roche	MA1-980-HRP	Mouse	IB	1:10000	Datasheet by company
HA-HRP	Roche	12013819001	Rat	IB	1:2000	Datasheet by company
FK2-HRP	Enzo	BML-PW0150	Mouse	IB	1:10000	Datasheet by company
HA	Biolegend	901501	Mouse	IP	1 μg	Datasheet by company
Flag	Sigma	F1804	Mouse	IB, IP	1:1000 for IB, 1μg for IP	Datasheet by company
β-actin	Sigma	a5316	Mouse	IB	1:10000	Datasheet by company
α-Tubulin	Millipore	05-829	Mouse	IB	1:5000	Datasheet by company
Cyp2e1	Abcam	ab28146	Rabbit	IB	1:1000	Datasheet by company

IB: immunoblot, IP: immunoprecipitation
